# Supplementary material for: Ru(II) photocages enable precise control over enzyme activity with red light
Source: Nat Commun. 2022 Jun 25;13:3636. doi: 10.1038/s41467-022-31269-5 (PMC9233675; doi:10.1038/s41467-022-31269-5)
Supplement: Supplementary file 1 — Supplementarty Information [file 41467_2022_31269_MOESM1_ESM.pdf]

Supplementary Information

Ru(II) photocages enable precise control over enzyme activity with red light

Dmytro Havrylyuk[1], Austin C. Hachey[1], Alexander Fenton[1], David K. Heidary[1*] & Edith C. Glazer[1*]

[1]Department of Chemistry, University of Kentucky, 505 Rose Street, Lexington, Kentucky 40506, United States

**Supplementary Methods**<br>**Synthesis**	S2–3
**Stability**	S3
**Kinetic solubility**<br>**Docking and MD experiments**	S4<br>S4–5
**Supplementary Figures**<br>**Supplementary Figure 1.** RMSD of bound ligand in CYP1B1 for compound **2** and **3**.	S6
**Supplementary Figure 2.** Absorption spectra of **4–6** in diH_2O.	S6
**Supplementary Figure 3–11.** Photoejection of **4–8.**	S7–11
**Supplementary Figures 12–17**. Stability of complexes **4–8**.	S12–15
**Supplementary Figures 18–25**. Dose responses in CYP1B1, 1A1, 19A1, phLMs and viability in HEK293 T-Rex cell line.	S16–21
**Supplementary Figure 26.** Thermal melt of recombinant CYP1B1.	S21
**Supplementary Figure 27.** Determination of irradiation-induced $^1O_2$.	S22
**Supplementary Figures 28–38**. NMR spectra of compounds **2**–**8**.	S22–27
**Supplementary Figures 39–43.** HRMS spectra of compounds **2**–**8**.	S28–32
**Supplementary Figure 44**. HPLC chromatograms of **6** with different detection wavelengths.	S33
**Supplementary Figure 45.** Relative Intensity of T-1 3/4 (5 mm) Solid State Lamp.<br>**Supplementary Figure 46.** Relative locations of residues enumerated in Supplementary Table 1.	S33<br>S34
**Supplementary Tables**<br>**Supplementary Table 1.** Prevalence of select protein-ligand contacts.	S34
**Supplementary Table 2.** IC_{50} values for inhibitors in different CYP1B1 variants. The ratio is the IC_{50} (mutant)/ IC_{50} (WT).	S35
**Supplementary Table 3.** Kinetic solubility test for compounds **4–6**.	S35
**Supplementary Table 4–6.** Stability of compounds in liver microsomes.	S35–36
**Supplementary Table 7.** Quantum yields of photosubstitution in different solvents.	S36
**Supplementary Table 8.** Lipinski's rule of five.	S36
**References**	S36

Supplementary Methods

Synthesis

4-(2,4-dimethoxystyryl)-pyrimidine (2). To a 38 mL pressure tube, 4-methylpyrimidine (0.3 g, 3.2 mmol) and 2,4-dimethoxy-benzaldehyde (3.5 mmol) were added, with 10 mL of methanol in aqueous sodium hydroxide (1.0 g in 5 mL of water). The mixture was stirred at 110°C for 2 hours, and then allowed to cool to room temperature. The yellow precipitate was filtered off, and washed with water and hexane. The purification of the solid was performed using flash chromatography (SiO_2, CH_3CN) to give the pure compound. Yield: 463 mg (60%). ^1H NMR (400 MHz, $CDCl_3$): δ 9.12 (s, 1H), 8.60 (d, J = 5.4 Hz, 1H), 8.13 (d, J = 16.2 Hz, 1H), 7.56 (d, J = 8.6 Hz, 1H), 7.32 (dd, J = 5.4, 1.2 Hz, 1H), 7.05 (d, J = 16.1 Hz, 1H), 6.54 (dd, J = 8.6, 2.4 Hz, 1H), 6.48 (d, J = 2.4 Hz, 1H), 3.90 (s, 3H), 3.85 (s, 3H). 1H NMR (400 MHz, CD_3OD): δ 8.99 (s, 1H), 8.61 (d, J = 5.4 Hz, 1H), 8.16 (d, J = 16.2 Hz, 1H), 7.61 (d, J = 9.2 Hz, 1H), 7.51 (d, J = 5.4 Hz, 1H), 7.10 (d, J = 16.2 Hz, 1H), 6.59-6.60 (m, 2H), 3.93 (s, 3H), 3.86 (s, 3H); 13C NMR (DMSO-D6): δ 162.29, 161.92, 158.97, 158.37, 157.41, 131.36, 128.92, 123.14, 118.77, 116.71, 106.02, 98.37, 55.64, 55.37. HRMS calcd for $C_{14}H_{14}N_2O_2$ $[MH]^+$ 243.1134, found 243.1128 $[MH]^+$. Elemental analysis calcd (%) for **2**: C, 69.41; H, 5.82; N, 11.56; found: C, 68.45; H, 5.71; N, 11.26.

4-(2,4-dimethoxystyryl)-pyridine (3). To a 25 mL round bottom flask 2,4-dimethoxybenzaldehyde (0.35 g, 2.2 mmol), 4-picoline (2.2 mmol), and potassium tert-butoxide (0.49 g, 4.4 mmol) were added, and dissolved in 10 mL of DMF. The reaction mixture was stirred at room temperature for 15 hours. It was then dried under vacuum and dissolved in 50 mL of water. The product was isolated by extraction into dichloromethane (100 mL) and the solvent was removed under reduced pressure. Purification of the solid was performed using flash chromatography (SiO_2, CH_3CN) to give the pure compound. Yield: 300 mg (69%). ^1H NMR (400 MHz, $CDCl_3$): δ 8.50 (d, J = 4.6 Hz, 2H), 7.57 (d, J = 16.2 Hz, 1H), 7.49 (d, J = 8.6 Hz, 1H), 7.32 (d, J = 4.6 Hz, 2H), 6.90 (d, J = 16.2 Hz, 1H), 6.50 (d, J = 8.8 Hz, 1H), 6.45 (s, 1H), 3.85 (s, 3H), 3.82 (s, 3H). ^{13}C NMR ($CDCl_3$): δ 161.52, 158.63, 150.12, 145.75, 128.08, 127.98, 124.08, 120.77, 118.35, 105.23, 98.51, 55.61, 55.54. HRMS calcd for $C_{15}H_{15}NO_2$ $[MH]^+$ 242.1181, found 242.1174 $[MH]^+$. Elemental analysis calcd (%) for **3**: C, 74.67; H, 6.27; N, 5.81; found: C, 74.39; H, 6.28; N, 5.85.

[Ru(tpy)(biq)Cl]PF$_6$ was synthesized as described previously[1] with the minor modification. The mixture of Ru(tpy)Cl_3 (200 mg, ~0.45 mmol; this compound may be a dimer) and biquinoline (biq, 116 mg, 0.45 mmol) were added to 5 mL of ethylene glycol in a pressure tube. The mixture was stirred at 180 °C for 1 hour, cooled to room temperature, diluted with EtOH (10 mL), and filtered over Celite. The filtrate was transferred into 100 mL of H_2O following the addition of saturated aqueous KPF_6 solution (1 mL) and extracted in CH_2Cl_2 (2 x 200 mL). The organic solvent was removed under reduced pressure and Et_2O was added to the residue. The purple solid was collected by vacuum filtration and washed with ether.

[Ru(tpy)(bca)Cl]PF$_6$. A mixture of Ru(tpy)Cl_3 (100 mg, ~0.23 mmol), bicinchoninic acid disodium salt (bca, 97 mg, 0.25 mmol), and LiCl (48 mg, 1.1 mmol) were added to 5 mL of ethylene glycol in a pressure tube. The mixture was stirred at 180 °C for 1 hour, cooled to room temperature, and transferred into 100 mL of H_2O. To this 2 mL of 1 M HCl and saturated aqueous KPF_6 solution (1 mL) were added, resulting in a precipitate which was collected by vacuum filtration and washed with ether.

[Ru(tpy)(biq)(2)]$^{2+}$ (4). [Ru(tpy)(biq)Cl]PF$_6$ (50 mg, 0.065 mmol) and 5-fold excess of **2** (78 mg, 0.325 mmol) were added to 6 mL of degassed EtOH : H_2O (5:1) in a pressure tube. The mixture was then stirred at 80 °C for 2 hours, before the reaction was stopped and the mixture transferred into 50 ml of H_2O. Addition of a saturated aqueous KPF_6 solution (ca. 1 mL) produced a red precipitate that was collected by vacuum filtration and washed with ether. Purification of the solid was performed using flash chromatography (SiO_2, 0.2% saturated KNO_3, 2% water in CH_3CN, ramped to 10% H_2O) to give the pure complex. After column purification, the isolated NO_3^- salt of the complex was dissolved in minimal water and converted to the PF_6^- salt upon the addition of a saturated solution of KPF_6. The precipitate was isolated by extraction into dichloromethane and the solvent was removed under reduced pressure. Yield: 30 mg (41%). Purity by HPLC = 92 %. This compound suffers from low thermal stability, particularly in coordinating solvents such as MeCN. Due to the mobile phase used in the HPLC, the isolated compound is anticipated to be higher purity than this calculated value. ^1H NMR (400 MHz, $(CD_3)_2CO$): δ 9.28 (d, J = 8.8 Hz, 1H), 9.16 (d, J = 8.8 Hz, 1H), 9.03 (d, J = 8.8 Hz, 1H), 8.37-8.98 (m, 9H), 8.07-8.20 (m, 4H), 7.96-7.99 (m, 2H), 7.83 (t, J = 7.7 Hz, 1H), 7.74 (d, J = 8.8, 1H), 7.51-7.61 (m, 5H), 7.40 (t, J = 8.7 Hz, 1H), 7.10 (d, J = 6.4 Hz, 1H), 6.93-6.99 (m, 2H), 6.53-6.58 (m, 2H), 3.87 (s, 3H), 3.83 (s, 3H). HRMS calcd for $C_{47}H_{37}N_7O_7Ru$ $[M]^{2+}$ 416.6026; found 416.6023 $[M]^{2+}$; UV/Vis (CH_3CN): λ_{max} ($\varepsilon \times 10^{-3}$) 535 nm (10.9). Elemental analysis calcd (%) for [**4**]2PF$_6$: C, 50.27; H, 3.32; N, 8.73; found: C, 50.48; H, 3.25; N, 8.49.

[Ru(tpy)(bca)(2)] (5). [Ru(tpy)(bca)Cl]PF_6 (100 mg, 0.12 mmol) and a 5-fold excess of **2** (0.6 mmol) were added to 10 mL of degassed EtOH : H_2O (4:1) in a pressure tube. The mixture was stirred at 80 °C for 15 hours. The reaction mixture then cooled to room temperature and solvents removed under reduced pressure. The purification of the solid was carried out by flash chromatography (SiO_2, 2% water in MeOH, ramped to 20% H_2O) to give the pure complex. The product fractions were concentrated under reduced pressure. Yield: 58 mg (40%). Purity by HPLC = 97 %. ^{1}H NMR (400 MHz, CD3OD): δ 9.16 (s, 1H), 8.90 (s, 1H), 8.55-8.85 (m, 5H), 8.37 (t, J = 8.0 Hz, 1H), 8.29 (d, J = 8.4 Hz, 1H), 8.01-8.15 (m, 6 H), 7.74 (t, J = 6.8 Hz, 1H), 7.65 (t, J = 6.4 Hz, 1H), 7.39-7.56 (m, 6H), 7.23 (t, J = 6.8 Hz, 1H), 7.03 (d, J = 6.2 Hz, 1H), 6.90 (d, J = 16.0 Hz, 1H), 6.76 (d, J = 8.8 Hz, 1H), 6.51-6.54 (m, 2H), 3.83 (s, 3H), 3.82 (s, 3H); ^{13}C NMR (100 MHz, MeOD) δ 171.72, 170.86, 164.43, 163.57, 160.18, 160.10, 159.26, 158.72, 158.40, 156.70, 151.26, 150.37, 149.91, 149.49, 138.88, 136.89, 136.61, 130.91, 130.65, 129.84, 128.72, 128.68, 128.17, 126.80, 126.49, 125.33, 123.09, 120.28, 118.67, 118.07, 116.54, 105.87, 97.71, 54.71, 54.60. HRMS calcd for $C_{49}H_{37}N_7O_6Ru$ $[M]^{2+}$ 460.5925; found 460.5926 $[M]^{2+}$; UV/Vis (H_2O): λ_{max} ($\varepsilon \times 10^{-3}$) 540 nm (6.3).

[Ru(tpy)(bca)(3)] (6). [Ru(tpy)(bca)Cl]PF_6 (35 mg, 0.042 mmol) and 5-fold excess of 3 (50 mg, 0.21 mmol) were added to 10 mL of degassed EtOH : H_2O (4:1) in a pressure tube. The mixture was stirred at 80 °C for 15 hours. The reaction mixture then cooled to room temperature and solvents removed under reduced pressure. The purification of the solid was carried out by flash chromatography (SiO_2, 2% water in MeOH, ramped to 20% H_2O) to give the pure complex. The product fractions were concentrated under reduced pressure and precipitated in 100 mL of acetonitrile : ether (1:9) to produce a purple precipitate that was collected by vacuum filtration and washed with ether. Yield: 21 mg (54%). Purity by HPLC = 99.5 %. ^{1}H NMR (400 MHz, CD_3OD): δ 9.17 (s, 1H), 8.91 (s, 1H), 8.80 (d, J = 8.6 Hz, 2H), 8.76 (d, J = 8.4 Hz, 1H), 8.62 (d, J = 8.0 Hz, 2H), 8.35 (t, J = 8.0 Hz, 1H), 8.28 (d, J = 8.4 Hz, 1H), 8.07-8.14 (m, 4 H), 7.71 (t, J = 8.0 Hz, 1H), 7.41-7.55 (m, 8H), 7.29 (t, J = 8.0 Hz, 1H), 7.21 (t, J = 8.0 Hz, 1H), 7.08 (d, J = 6.2 Hz, 2H), 6.84 (d, J = 16.4 Hz, 1H), 6.76 (d, J = 8.8 Hz, 1H), 6.45-6.50 (m, 2H), 3.82 (s, 3H), 3.80 (s, 3H); ^{13}C NMR (100 MHz, CD_3OD) δ 171.78, 170.94, 162.61, 160.04, 159.26, 159.13, 158.42, 158.32, 153.11, 151.28, 150.46, 150.15, 150.08, 149.28, 148.61, 138.70, 136.59, 131.69, 130.59, 130.50, 128.68, 128.59, 128.46, 128.13, 126.73, 126.41, 125.29, 124.64, 123.84, 123.24, 121.92, 120.76, 118.09, 117.01, 105.51, 97.69, 54.64, 54.51. HRMS calcd for $C_{50}H_{38}N_6O_6Ru$ $[M]^{2+}$ 460.0948; found 460.0958 $[M]^{2+}$; UV/Vis (H_2O): λ_{max} ($\varepsilon \times 10^{-3}$) 550 nm (6.6). Elemental analysis calcd (%) for [**6**]$2PF_6$•$2CH_3CN$•$3C_4H_{10}O$: C, 52.35; H, 4.93; N, 7.40; found: C, 52.71; H, 4.35; N, 7.37.

[Ru(tpy)(bca)(pyridine)] (8). [Ru(tpy)(bca)Cl]PF_6 (80 mg, 0.096 mmol) and 1 mL of pyridine were added to 5 mL of degassed EtOH : H_2O (4:1) in a pressure tube. The mixture was stirred at 80°C for 3 hours. The reaction mixture then cooled to room temperature and solvents removed under reduced pressure. The purification of the solid was carried out by flash chromatography (SiO_2, 2% water in MeOH, ramped to 30% H_2O) to give the pure complex. The product fractions were concentrated under reduced pressure and precipitated in 100 mL of acetonitrile : ether (1:9) to produce a purple precipitate that was collected by vacuum filtration and washed with ether. Yield: 53 mg (65%). Purity by HPLC = 99.0 %. ^{1}H NMR (400 MHz, (CD_3OD): δ 9.14 (s, 1H), 8.85 (s, 1H), 8.78 (d, J = 8.1 Hz, 2H), 8.73 (d, J = 8.4 Hz, 1H), 8.60 (d, J = 8.0 Hz, 2H), 8.34 (t, J = 8.0 Hz, 1H), 8.26 (d, J = 8.4 Hz, 1H), 8.12 (d, J = 5.6 Hz, 2H), 8.08 (t, J = 7.8 Hz, 2H), 7.66-7.71 (m, 4H), 7.50 (t, J = 6.4 Hz, 2H), 7.40 (t, J = 7.2 Hz, 1H), 7.30 (d, J = 8.8 Hz, 1H), 7.19-7.23 (m, 2H), 7.05 (t, J = 6.6 Hz, 2H), 6.73 (d, J = 8.4 Hz, 1H). ^{13}C NMR (100 MHz, CD_3OD) δ 173.08, 172.24, 161.51, 160.60, 159.83, 159.69, 154.56, 152.65, 151.65, 151.44, 150.82, 140.16, 139.80, 138.10, 132.01, 131.84, 130.03, 129.94, 129.88, 129.52, 128.16, 127.49, 127.31, 126.69, 126.07, 125.29, 124.58, 119.45. HRMS calcd for $C_{40}H_{28}N_6O_4Ru$ $[M]^{2+}$ 379.0608; found 379.0611 $[M]^{2+}$; λ_{max} (ε × 10-3) 540 nm (8.2). Elemental analysis calcd (%) for [**8**]PF_6•$2CH_3CN$•$2C_4H_{10}O$: C, 55.12; H, 4.80; N, 9.89; found: C, 55.05; H, 4.51; N, 9.57.

Aqueous Stability

Measured by UV/Vis: The aqueous stability of complexes **4–6** was studied at 37 °C as 50 μM solutions in di-H_2O, and in Opti-MEM™ with 2% fetal bovine serum. Each solution was measured in triplicate in a 96-well plate and monitored by UV/vis absorbance over the course of 24 hours. For compound **6**, the UV/Vis in aqueous solutions of the product was very similar to the starting material.

Measured by HPLC: Compounds **4** and **5** were diluted in water to 50 μM solutions. Compound **6** was prepared as a 40 μM solution in a mixture of di-H_2O : MeCN (4:1); MeCN was added to reduce the aggregation. The HPLC chromatograms were recorded at t=0, and the samples were incubated at 37 °C for 24 hours. Chromatograms were recorded for each compound.

Kinetic solubility was measured by UV/Vis. In this experiment, 14 µL of 10 mM DMSO stocks of complexes **4–6** were diluted in 700 µL of Opti-MEM™ with 2% fetal bovine serum and kept at room temperature for 3 hours. The solutions were then filtered using syringe-driven filters (FV12S) and concentrations were measured by UV/vis absorbance in a 96-well plate in triplicate.

Docking and MD Experiments

The procedures for the experiments are provided in the Methods section. Additional details for the experimental parameters are as follows:

Relaxation

The stages in the default relaxation process for the NPT ensemble are:
1. Minimize with the solute restrained
2. Minimize without restraints
3. Simulate in the NVT ensemble using a Berendsen thermostat with:
    - a simulation time of 12ps
    - a temperature of 10K
    - a fast temperature relaxation constant
    - velocity resampling every 1ps
    - non-hydrogen solute atoms restrained
4. Simulate in the NPT ensemble using a Berendsen thermostat and a Berendsen barostat with:
    - a simulation time of 12ps
    - a temperature of 10K and a pressure of 1 atm
    - a fast temperature relaxation constant
    - a slow pressure relaxation constant
    - velocity resampling every 1ps
    - non-hydrogen solute atoms restrained
5. Simulate in the NPT ensemble using a Berendsen thermostat and a Berendsen barostat with:
    - a simulation time of 24ps
    - a temperature of 300K and a pressure of 1 atm
    - a fast temperature relaxation constant
    - a slow pressure relaxation constant
    - velocity resampling every 1ps
    - non-hydrogen solute atoms restrained
6. Simulate in the NPT ensemble using a Berendsen thermostat and a Berendsen barostat with:
    - a simulation time of 24ps
    - a temperature of 300K and a pressure of 1 atm
    - a fast temperature relaxation constant
    - a normal pressure relaxation constant

Details Taken from Desmond User Manual: https://www.schrodinger.com/documentation

Simulation
Simulation Time (ns): 50
Recording Interval (ps): 50
Approximate Number of Frames: 1,000
Energy: 1.2
Ensemble Class: NPT
Temperature (K): 300
Pressure (bar): 1.01325

RESPA integrator
- Time step (fs):
  - Bonded: 2.00
  - Near: 2.00
  - Far: 6.00

Thermostat
- Method: Nose-Hoover chain
- Relaxation Time (ps): 1.0
- Number of Groups: 1

Barostat
- Method: Martyna-Tobias-Klein
- Relaxation time (ps): 2.0
- Coupling style: Isotropic

Interaction
- Coulombic
  - Short range method: Cutoff
  - Cutoff radius (Å): 9.0

Other

Protein Preparation: Charge states generated at pH 7 ± 2
Ligand Preparation: Charge states generated at pH 7 ± 2 using Epik
Glide Docking Precision: XP

Supplementary Figures

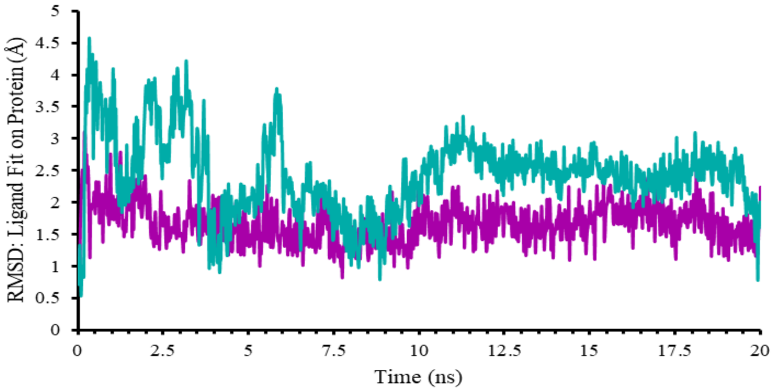

Supplementary Figure 1. RMSD of bound ligand in CYP1B1 for compound **2** (cyan) and **3** (magenta) over 20 ns molecular dynamics trajectory.

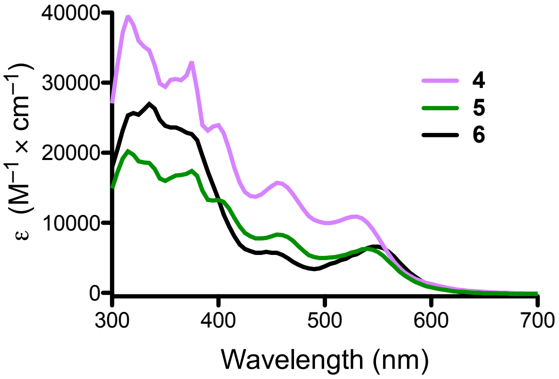

Supplementary Figure 2. Absorption spectra of **4**-**6** in di-H_2O.

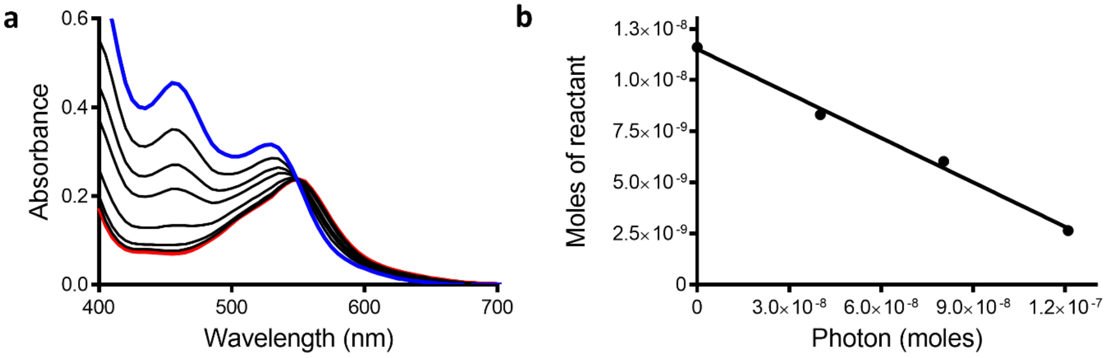

Supplementary Figure 3. Photoejection of **4** (50 μM, in di H_2O) over 0–3 min irradiation. The experiment was performed in triplicate (n = 3), and the data for one replicate are presented. **a** Time course from 0 (blue) to 3 (red) min, followed by UV/vis absorption. **b** Liner regression for moles of reactant vs. moles of photons absorbed for complex **4**. The light source was a 470 nm LED array (Elixa).

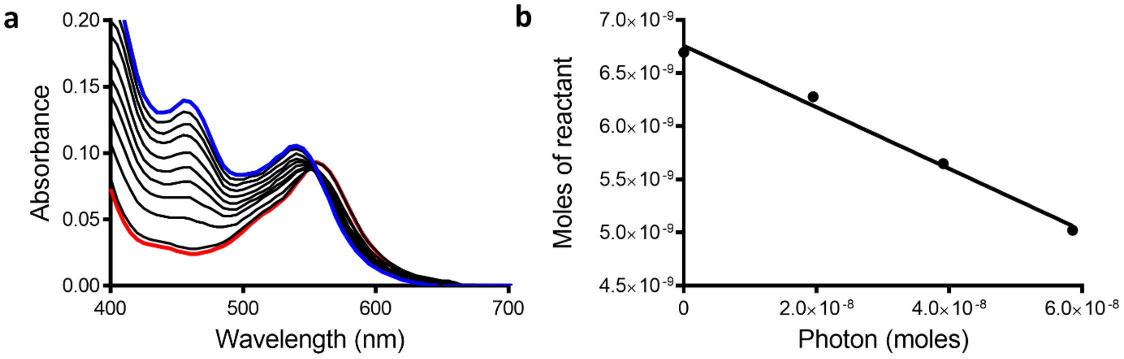

Supplementary Figure 4. Photoejection of **5** (30 μM, in diH_2O) for 0–10 min irradiation. The experiment was performed in triplicate (n = 3), the data for one replicate are presented. **a** Time course from 0 (blue) to 10 (red) min, followed by UV/vis absorption. **b** Liner regression for moles of reactant vs. moles of photons absorbed for complex **5.** The light source was a 470 nm LED array (Elixa).

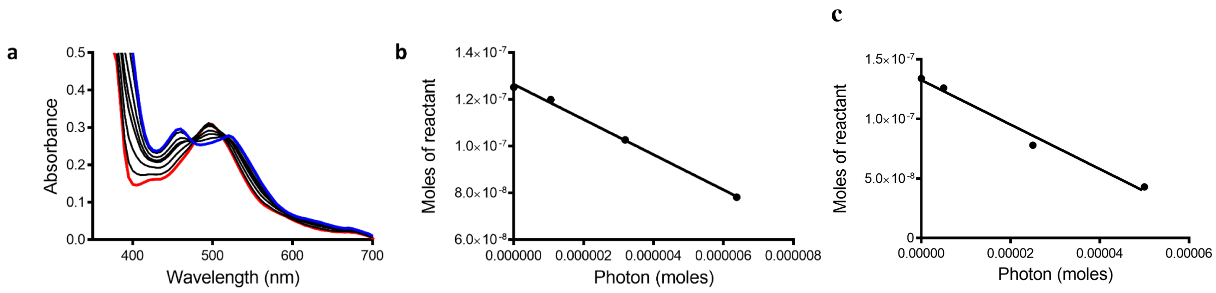

Supplementary Figure 5. Photoejection of **6** for 0–10 min irradiation in a quartz cuvette at a final concentration of 40 μM and a path length of 1 cm. The photon flux of the lamp for irradiation in the cuvette was determined by ferrioxalate actinometer (2.32E-7 Mol/s). The experiment was performed in triplicate (n = 3), the data for one replicate are presented. **a** Time course from 0 (blue) to 10 (red) min, followed by UV/vis absorption in MeCN. **b** Liner regression for moles of reactant vs. moles of photons absorbed for complex **6** in MeCN. **c** Liner regression for moles of reactant vs. moles of photons absorbed for complex **6** in MeOH. The light source was a 470 nm LED array (Elixa).

a

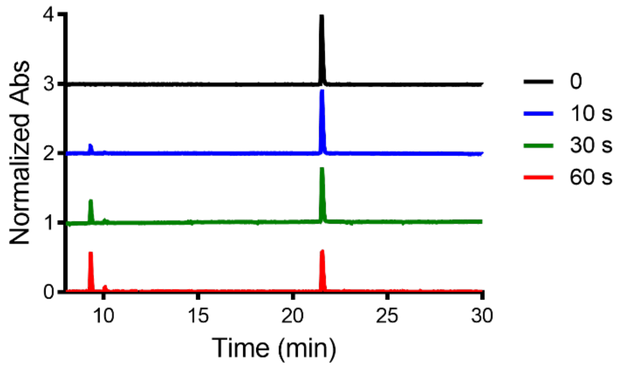

b

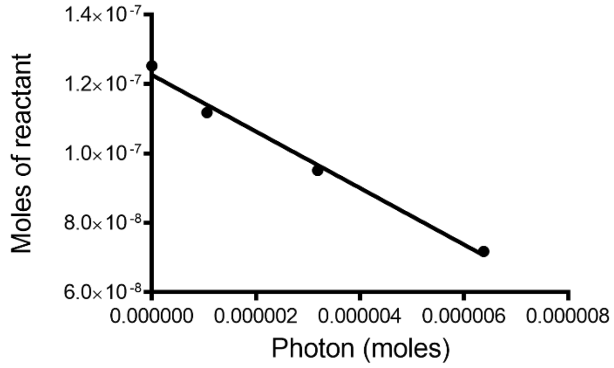

Supplementary Figure 6. Photoejection of **6** (40 μM, in MeCN) for 0–60 s irradiation in a quartz cuvette. **a** HPLC chromatogram analysis of photochemistry of **6** (40 μM, in MeCN) before irradiation (black line) and after irradiation for 10–60 s (detection wavelength = 280 nm). **b** Liner regression for moles of reactant vs. moles of photons absorbed for complex **6**. The light source was a 470 nm LED array (Elixa).

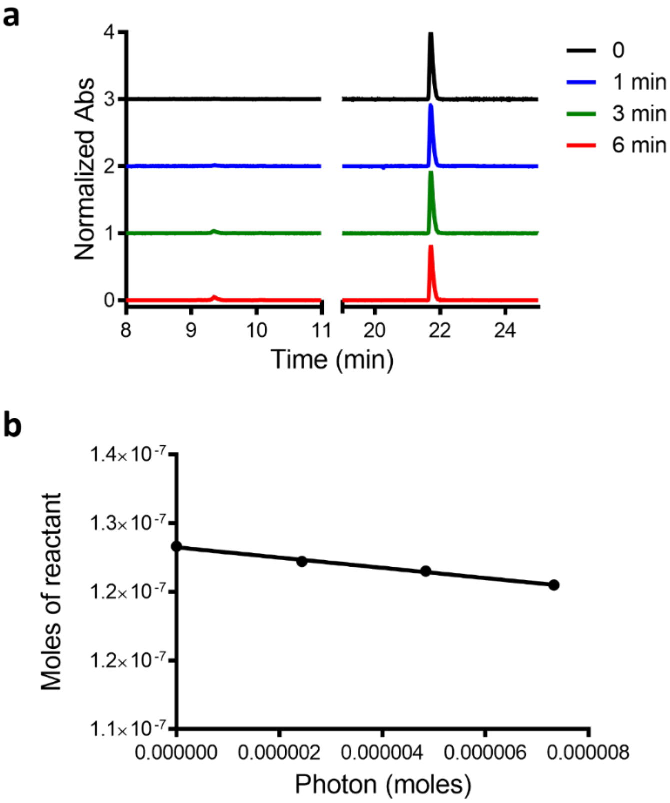

Supplementary Figure 7. Photoejection of **6** for 0–6 min irradiation in a quartz cuvette at a final concentration of 40 μM in 5% DMSO and a path length of 1 cm. The photon flux of the lamp for irradiation in cuvette was determined by ferrioxalate actinometer (2.32E-7 Mol/s). **a** HPLC chromatogram analysis of photochemistry of **6** before irradiation (black line) and after irradiation for 1, 3 and 6 min (detection wavelength = 280 nm). **b** Liner regression for moles of reactant vs. moles of photons absorbed for complex **6**. The light source was a 470 nm LED array (Elixa).

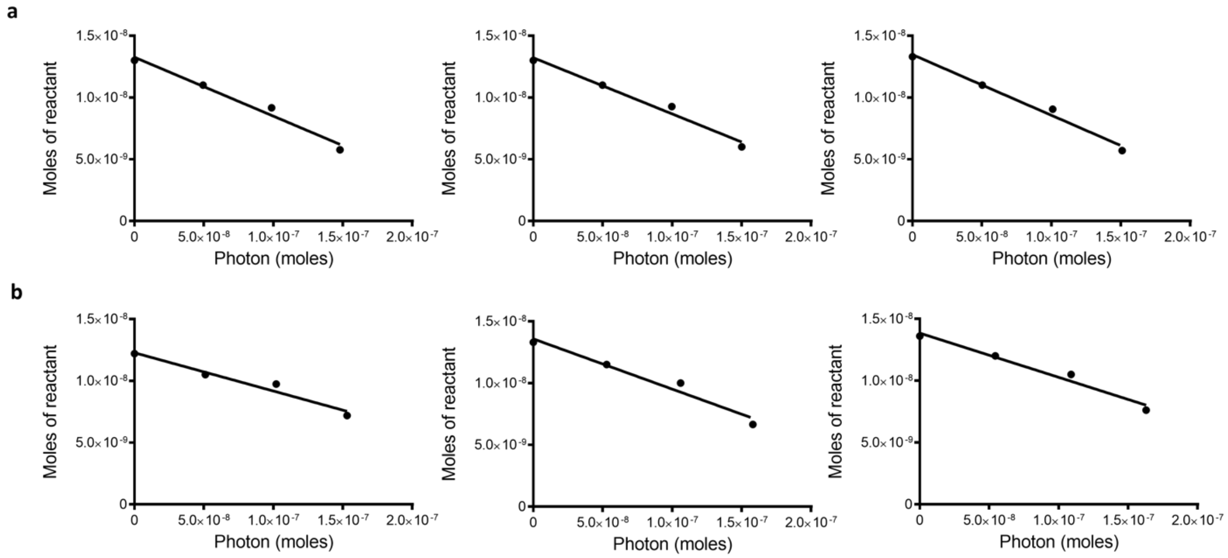

Supplementary Figure 8. Liner regression for moles of reactant vs. moles of photons absorbed for complex **4** (50 μM) in **a** 5% DMSO and **b** Opti-MEM. The compound was tested in triplicate (n = 3) for each condition. The light source was a 470 nm LED array (Elixa).

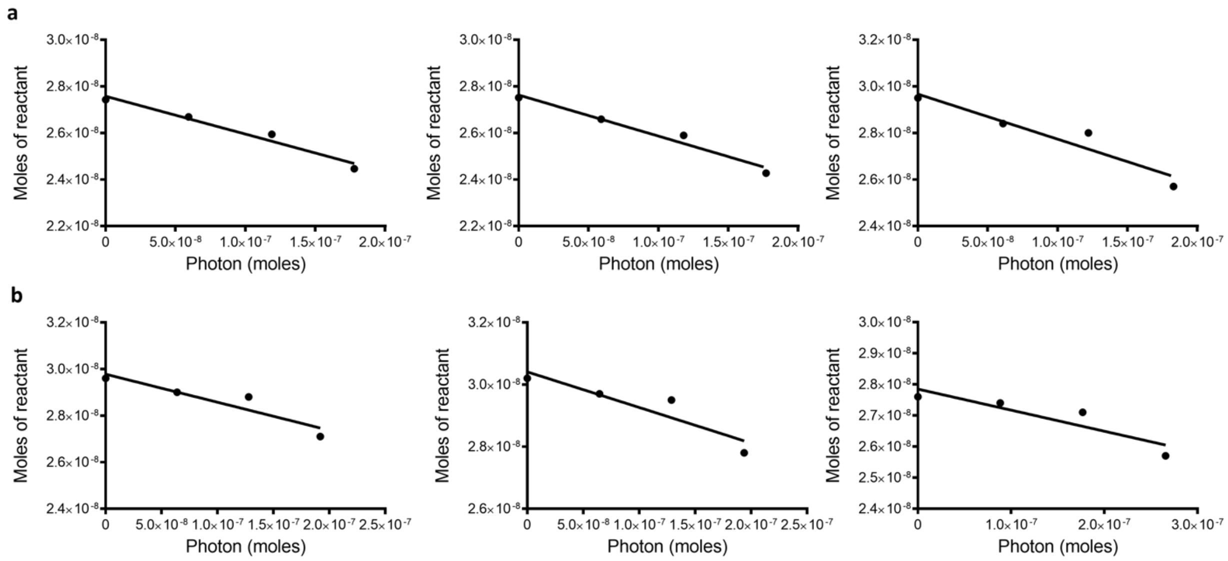

Supplementary Figure 9. Liner regression for moles of reactant vs. moles of photons absorbed for complex **5** (100 μM) in **a** 5% DMSO (A) and **b** Opti-MEM. The compound was tested in triplicate (n = 3) for each condition. The light source was a 470 nm LED array (Elixa).

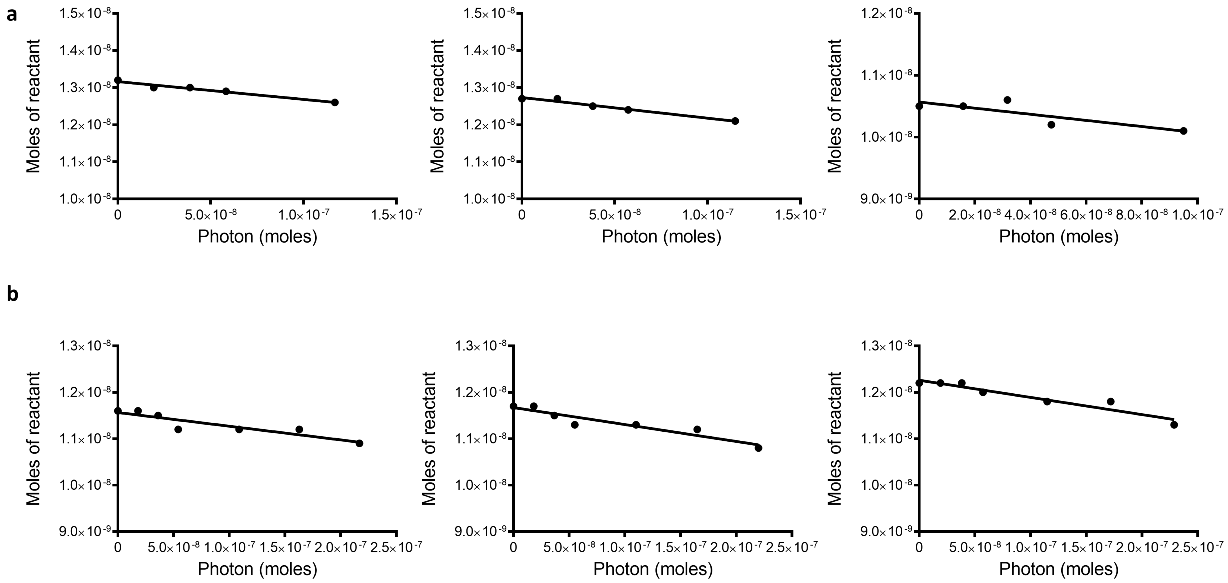

Supplementary Figure 10. Liner regression for moles of reactant vs. moles of photons absorbed for complex **8** [**Ru(tpy)(bca)(pyridine)**] (50 μM) in **a** 5% DMSO and **b** Opti-MEM. The compound was tested in triplicate (n = 3) for each condition. The light source was a 470 nm LED array (Elixa).

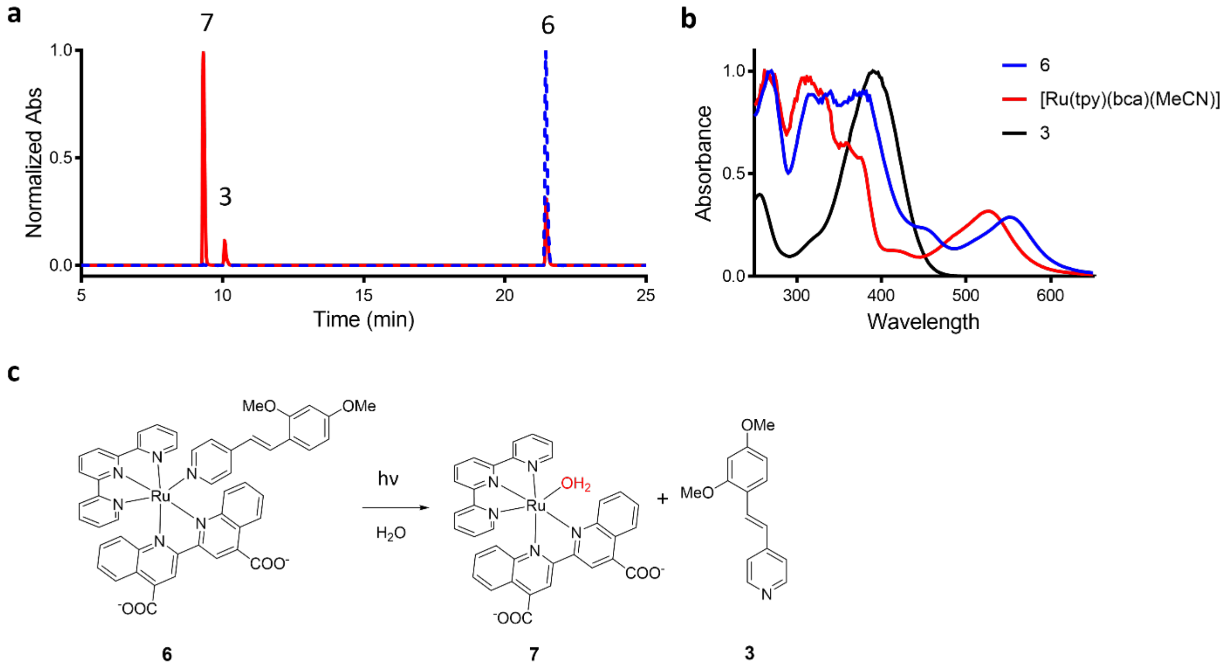

Supplementary Figure 11. **a** HPLC chromatogram analysis of photochemistry of **6** (100 μM in di H_2O : MeCN mixture (4:1), MeCN has been added to reduce aggregation) before (blue dashed line) and after 1 hour irradiation with red light (66o nm, 58.7 J/cm^2). **b** Absorption profile of **6** (blue, retention time = 21.5 min) and the photochemical products [Ru(tpy)(bca)(L)] (red, retention time = 9.3 min; L is most likely MeCN) and **3** (black, retention time = 10.0 min). Note that the presence of CH_3CN and 0.1% of formic acid in the HPLC experiment changes the absorption profile for the photoproducts. **c** Photoejection scheme for **6** in water**.**

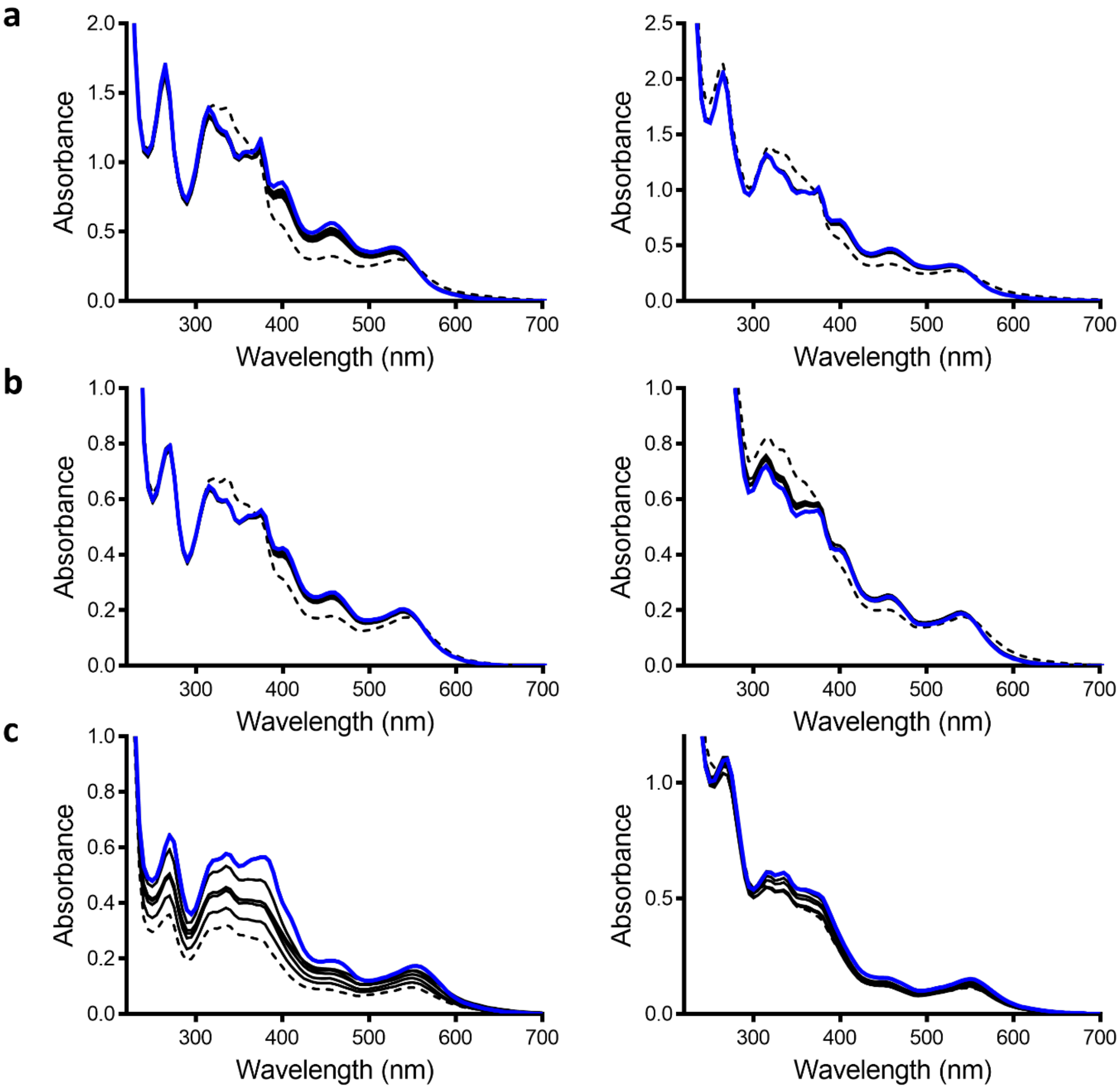

Supplementary Figure 12. Stability of complexes **4**–**6** (50–60 μM). **a** Complex **4**, **b 5**, and **c 6** in di-H_2O (left) and Opti-MEM with 2% fetal bovine serum (right) at 37 °C in the dark over the course of 0 (blue line) to 24 h (black dashed line). Compounds were tested in triplicate (n = 3), the data for one replicate are presented. Complexes **4** and **5** slowly degraded in the experiment condition (see HPLC traces, Figures S13–14). The decrease in absorbance for complex **6** is explained by the slow aggregation of the compound in the 96-well plate.

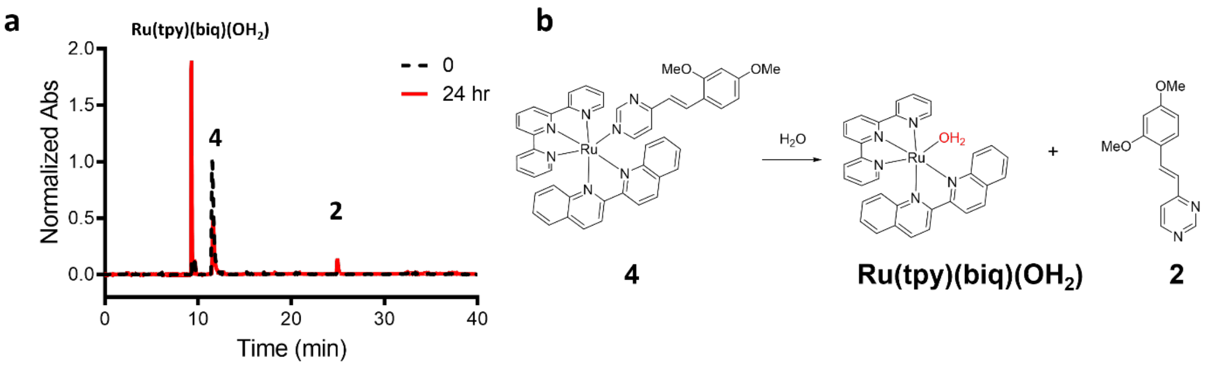

Supplementary Figure 13. a HPLC chromatogram analysis of stability of **4** (50 μM in di-H_2O) before (black dashed line, purity > 92 %) and after incubation at 37 °C for 24 hours (red line). At 24 hours, 43 % of **4** was observed remaining (calculated by area, detection wavelength = 280 nm). **b** Scheme of hydrolysis of compound **4**.

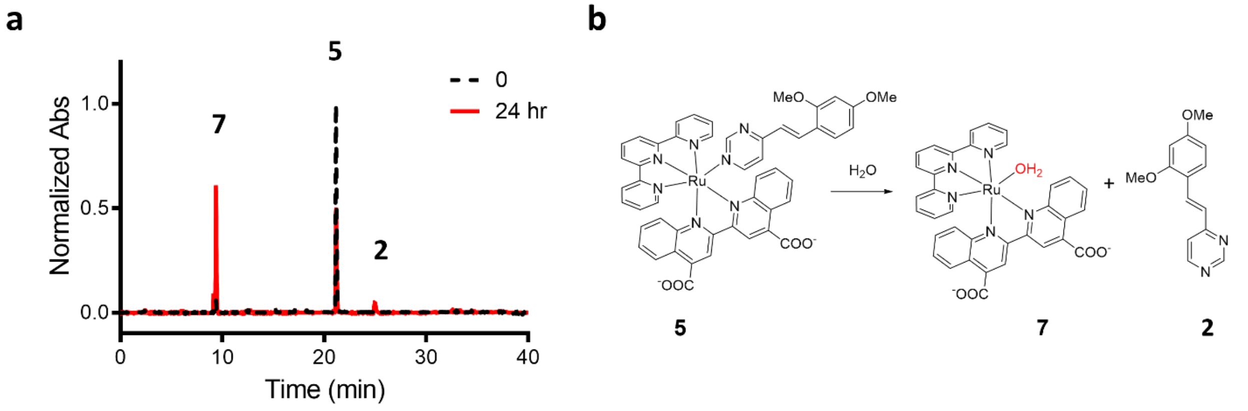

Supplementary Figure 14. HPLC chromatogram analysis of stability of **5** (50 μM in di-H_2O) before (black dashed line, purity > 97 %) and after incubation at 37 °C for 24 hours (red line). At 24 hours, 50 % of **5** was observed remaining (calculated by area, detection wavelength = 280 nm). **b** Scheme of hydrolysis of compound **5**.

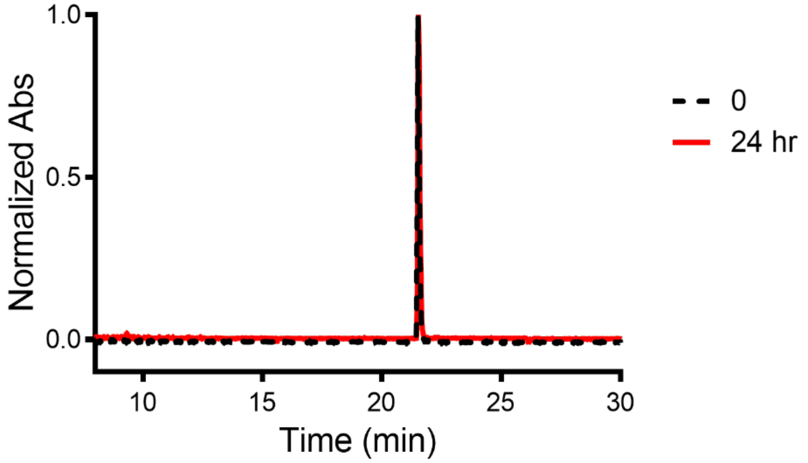

Supplementary Figure 15. HPLC chromatogram analysis of stability of **6** (40 μM in di-H_2O : MeCN mixture (4:1), MeCN was added to reduce the aggregation) before (black dashed line, purity > 99.5 %) and after incubation at 37 °C for 24 hours. At 24 hours, less than 2 % degradation was observed (calculated by area, detection wavelength = 280 nm).

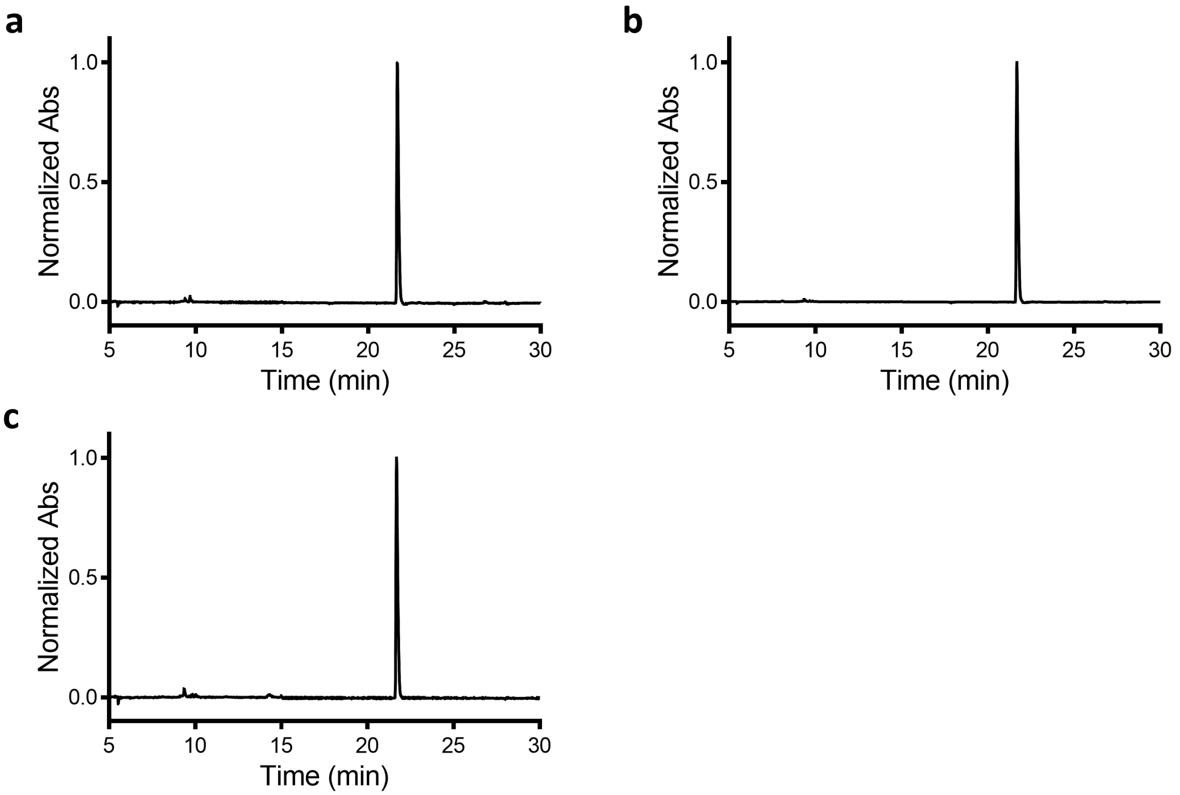

Supplementary Figure 16. HPLC chromatogram analysis of stability of **6** (40 μM) in 5 % DMSO / di-H_2O with **a** 100 μM GSH, **b** 100 μM imidazole and **c** acidic condition (pH = 1) after incubation at 37 °C for 72 hours. Less than 2 % degradation was observed in solutions with GSH or imidazole, and < 7 % at pH = 1 (calculated by area, detection wavelength = 280 nm).

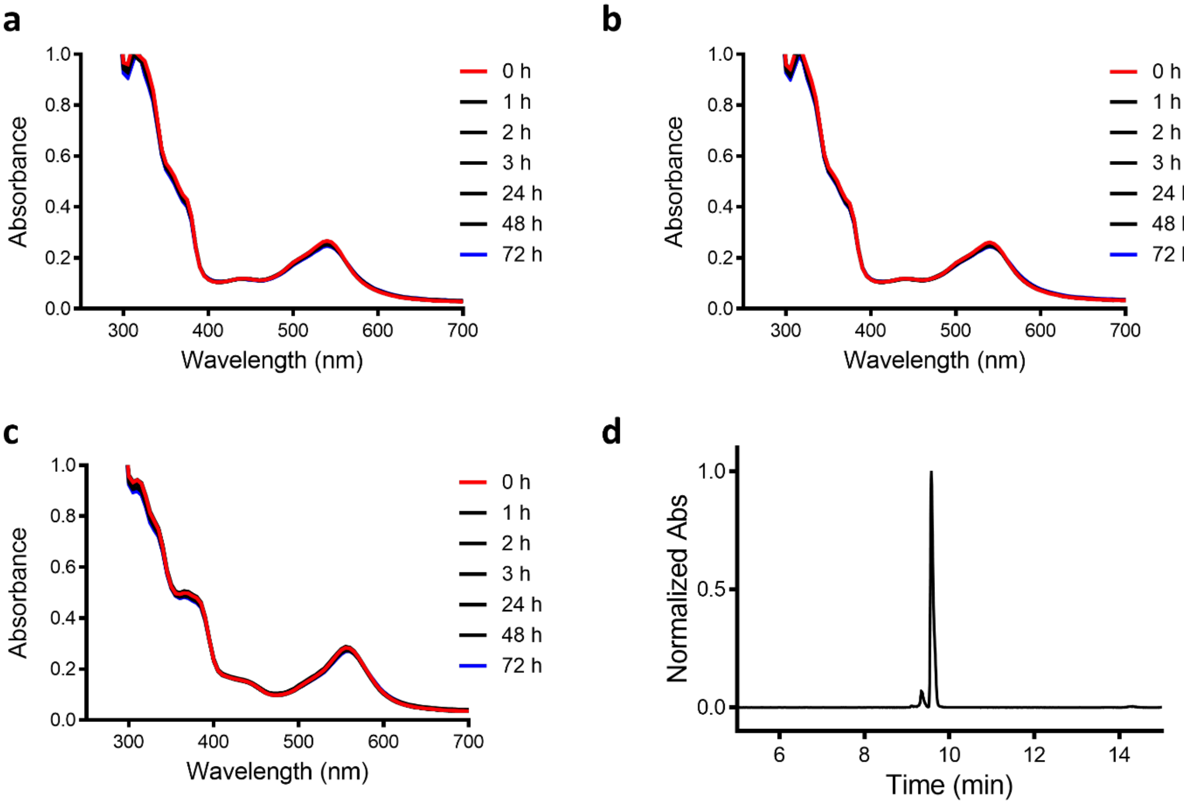

Supplementary Figure 17. Stability of complex **8** (50 μM) in 5 % DMSO / di-H_2O with **a** 100 µM GSH, **b** 100 µM imidazole and **c** acidic condition (pH = 1) after incubation at 37 °C for 72 hours. **d** At 72 hours less than 7 % degradation was observed by HPLC at pH = 1 (calculated by area, detection wavelength = 280 nm). Compounds were tested in triplicate (n = 3), the data for one replicate are presented.

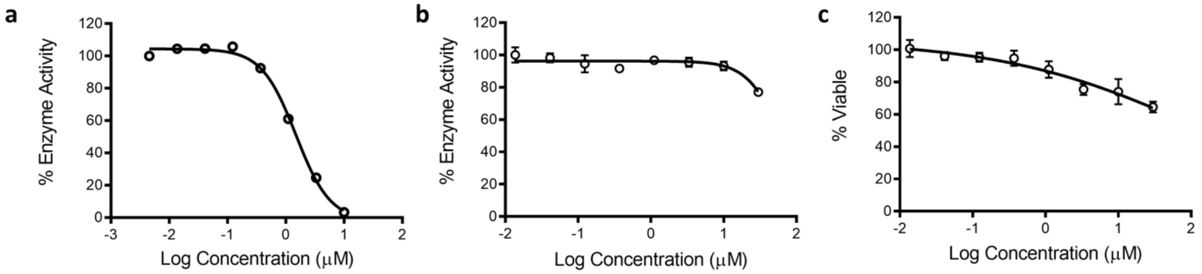

Supplementary Figure 18. Dose responses for **1** for inhibition of enzyme activity in **a** CYP1A1, **b** 19A1, and **c** viability in HEK293 T-Rex cell line (after 72 hrs). The error bars correspond to the standard deviation of the three replicates.

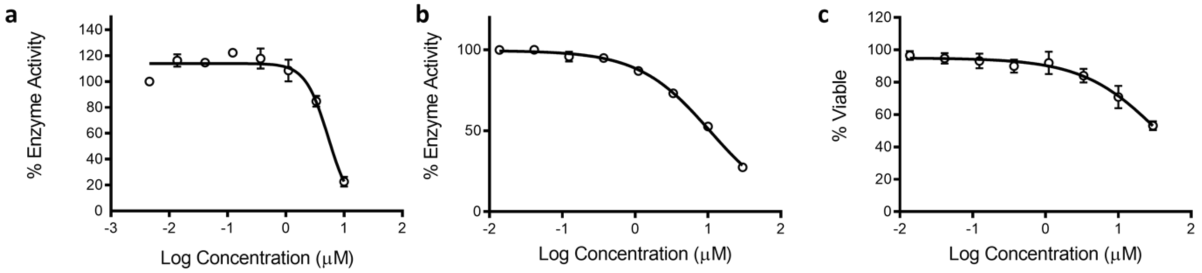

Supplementary Figure 19. Dose responses for **2** for inhibition of enzyme activity in **a** CYP1A1, **b** 19A1, and **c** viability in HEK293 T-Rex cell line (after 72 hrs). The error bars correspond to the standard deviation of the three replicates.

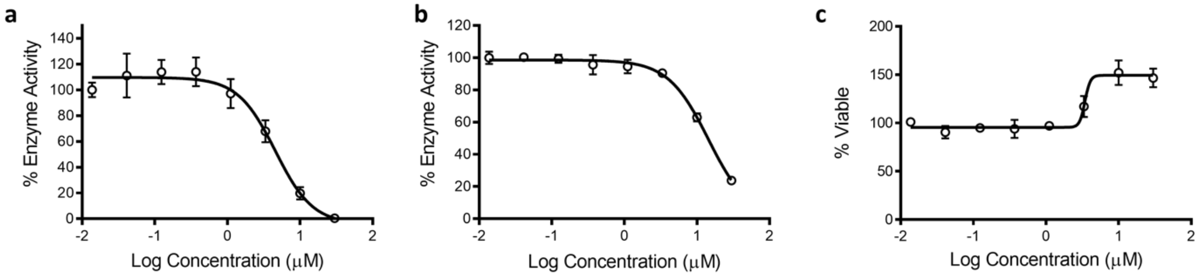

Supplementary Figure 20. Dose responses for **3** for inhibition of enzyme activity in **a** CYP1A1, **b** 19A1, and **c** viability in HEK293 T-Rex cell line (after 72 hrs). The error bars correspond to the standard deviation of the three replicates

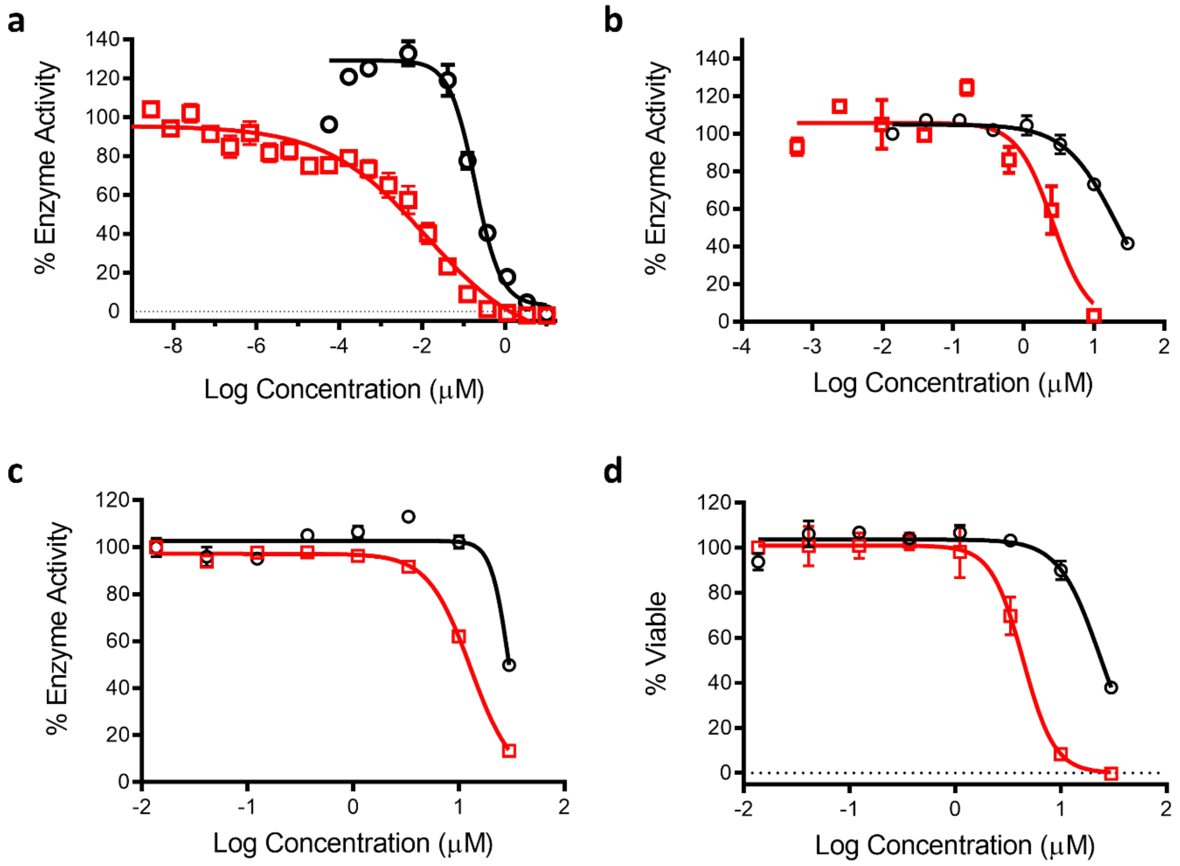

Supplementary Figure 21. Dose responses for **4** for inhibition of enzyme activity in **a** CYP1B1, **b** 1A1, **c** 19A1, and **d** viability in HEK293 T-Rex cell line (after 72 hrs). The error bars correspond to the standard deviation of the three replicates. Data taken in the dark are shown with black circles, and data taken following irradiation with 660 nm light (1 hr = 58.7 J/cm^2) is shown with red squares.

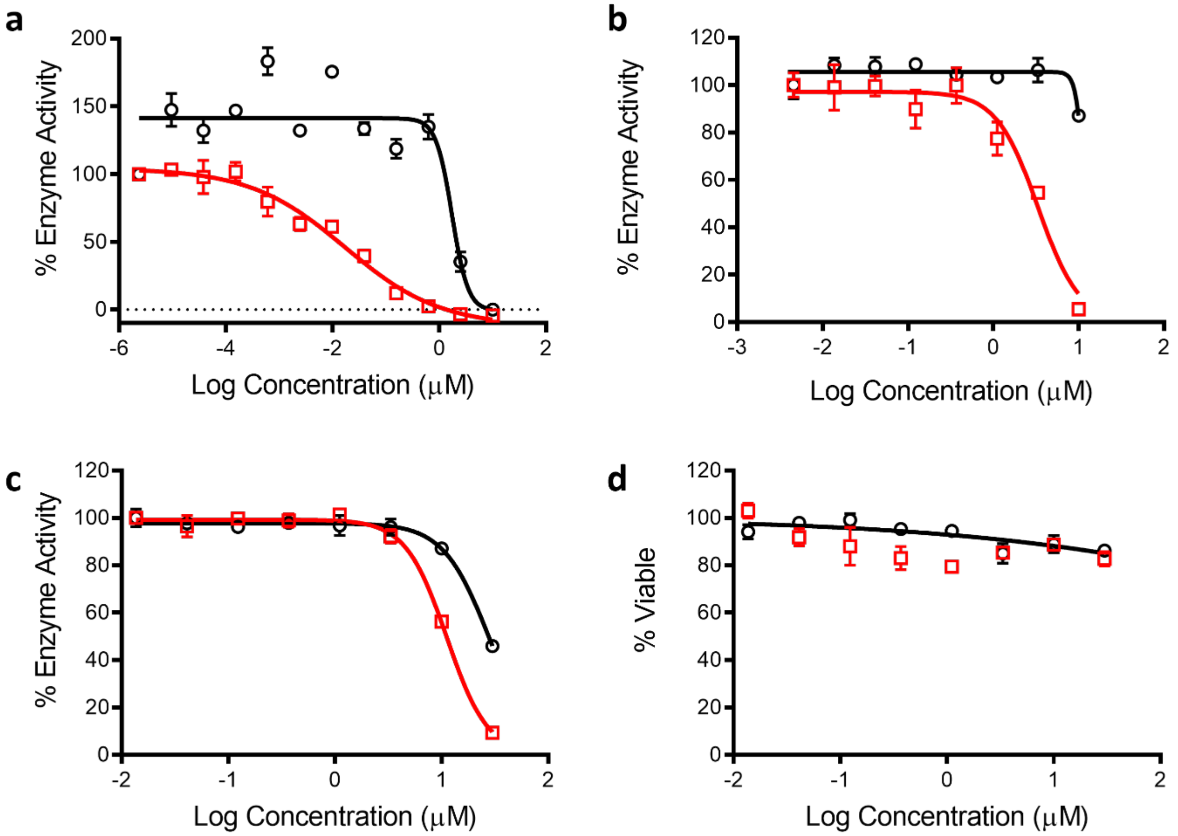

Supplementary Figure 22. Dose responses for **5** for inhibition of enzyme activity in **a** CYP1B1, **b** 1A1, **c** 19A1, and **d** viability in HEK293 T-Rex cell line (after 72 hrs). The error bars correspond to the standard deviation of the three replicates. Data taken in the dark are shown with black circles, and data taken following irradiation with 660 nm light (1 hr = 58.7 J/cm^2) is shown with red squares.

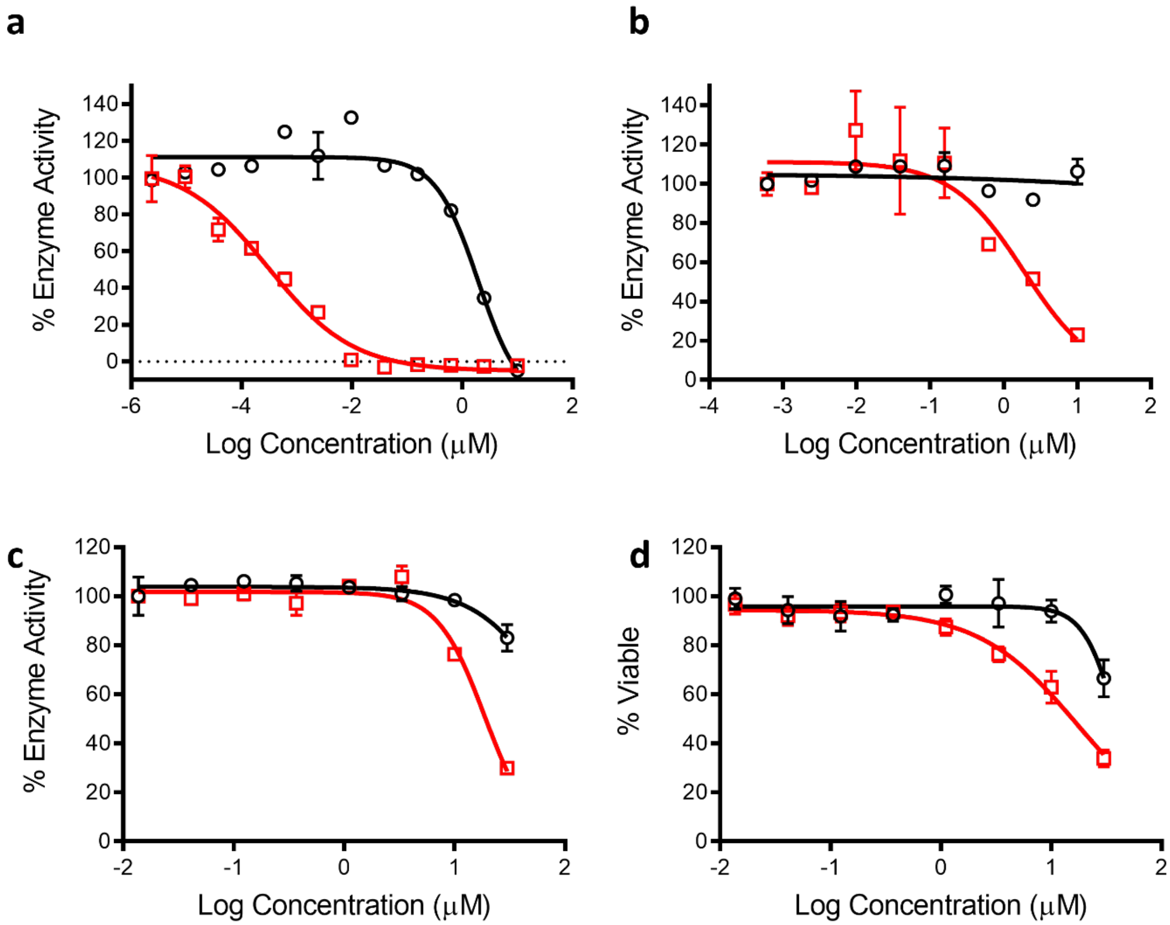

Supplementary Figure 23. Dose responses for **6** for inhibition of enzyme activity in **a** CYP1B1, **b** 1A1, **c** 19A1, and **d** viability in HEK293 T-Rex cell line (after 72 hrs). The error bars correspond to the standard deviation of the three replicates. Data taken in the dark are shown with black circles, and data taken following irradiation with 660 nm light (1 hr = 58.7 J/cm^2) is shown with red squares.

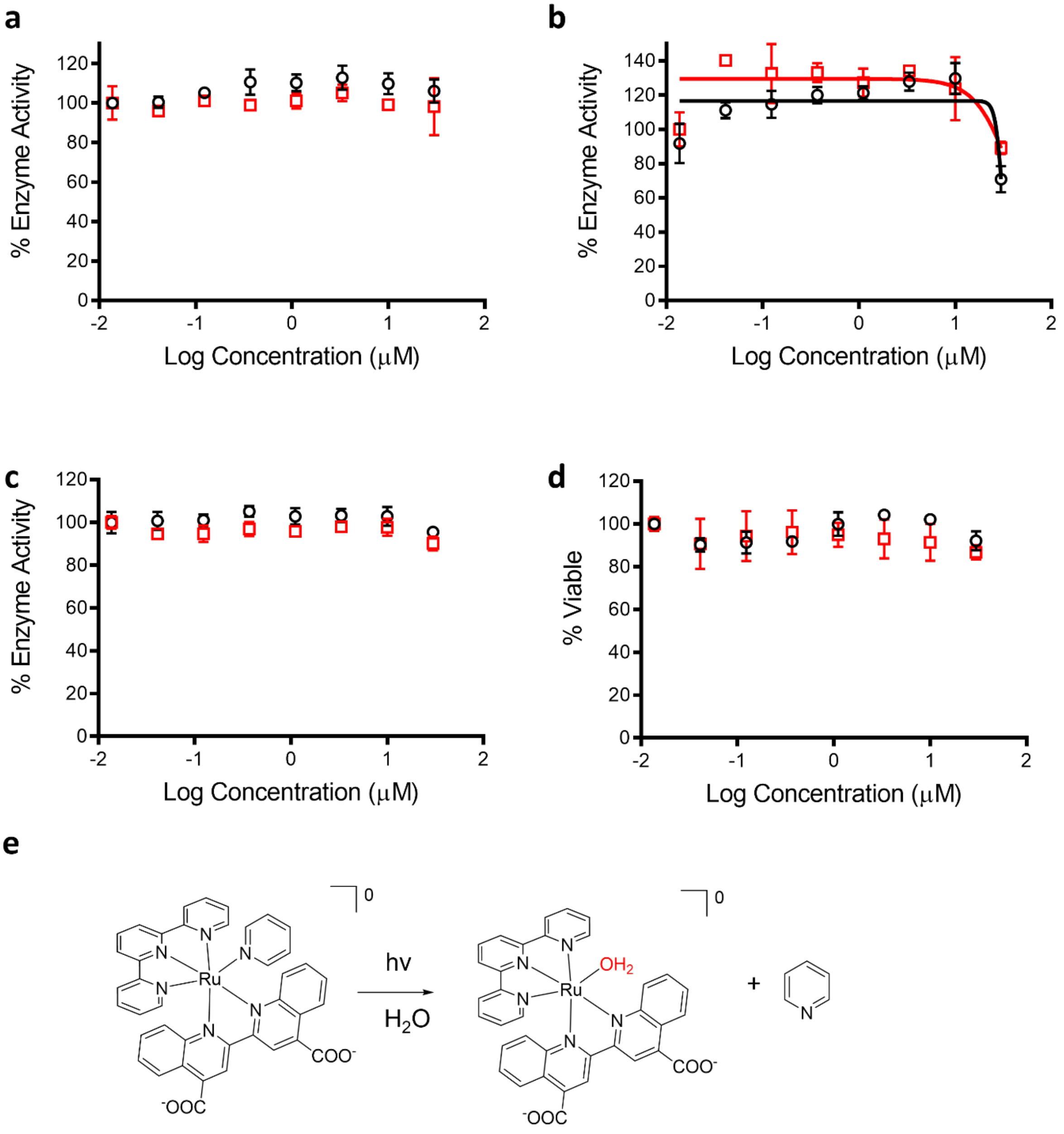

8 **7**

Supplementary Figure 24. Dose responses for **8** for inhibition of enzyme activity in **a** CYP1B1, **b** 1A1, **c** 19A1, and **d** viability in HEK293 T-Rex cell line (after 72 hrs). The error bars correspond to the standard deviation of the three replicates. Data taken in the dark are shown with black circles, and data taken following irradiation with 660 nm light (1 hr = 58.7 J/cm^2) is shown with red squares. **e** Photoejection scheme for **8** in water produces the same photoproduct, complex **7**, as is generated by compound **6**.

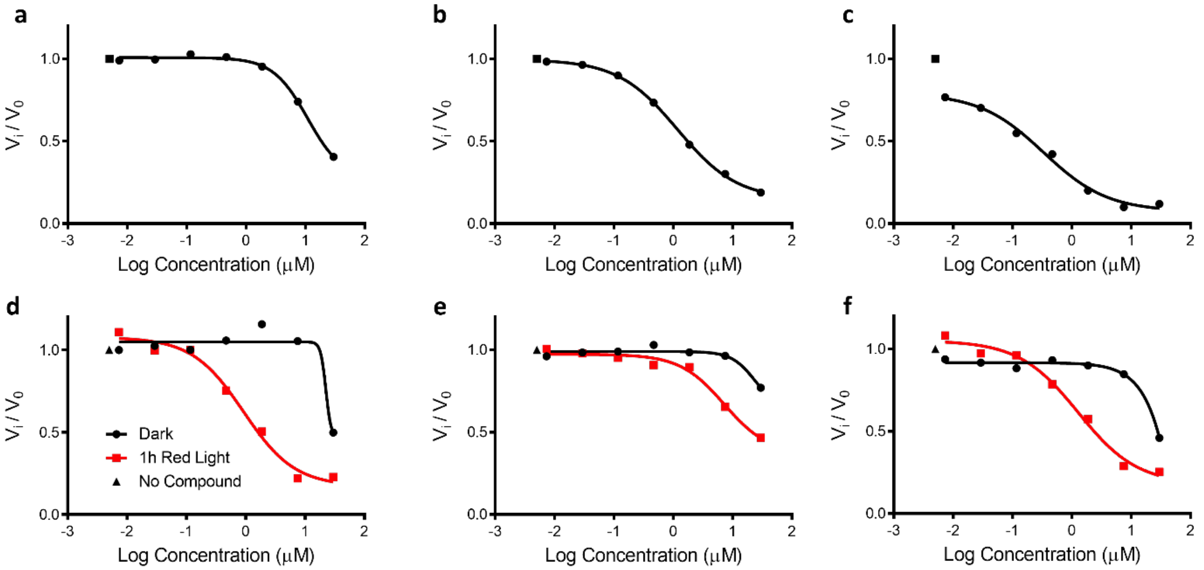

Supplementary Figure 25. Dose responses in phLMs for **a 1**, **b 2**, **c 3**, **d 4**, **e 5**, and **f 6**. The error bars correspond to the standard deviation of the three replicates. For **4–6**, data taken in the dark are shown with black circles, and data taken following irradiation with 660 nm light (1 hr = 58.7 J/cm^2) is shown with red squares.

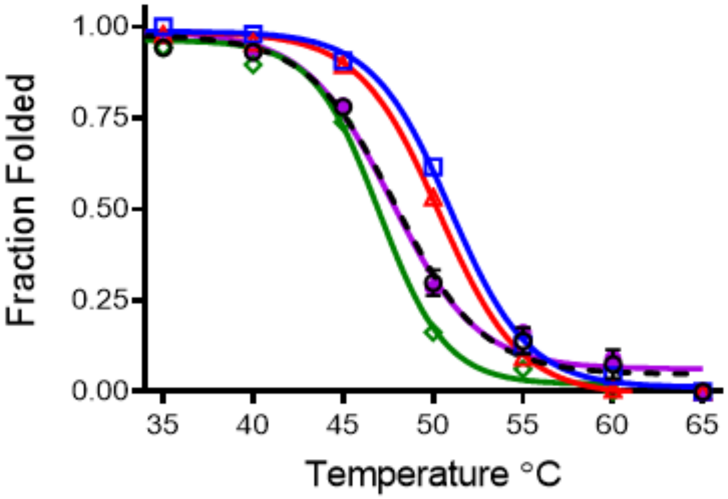

Supplementary Figure 26. Thermal melt of recombinant CYP1B1. Black circles dash line, no compound; blue squares, 10 μM ANF; red triangles, 10 μM **3**; green rhombs, 20 μM **6**; purple squares, 20 μM **8** following by irradiation with 660 nm light (1 hr = 58.7 J/cm^2). See Table 3 for T_m values from fits. The error bars for no compound correspond to the standard deviation of the three replicates.

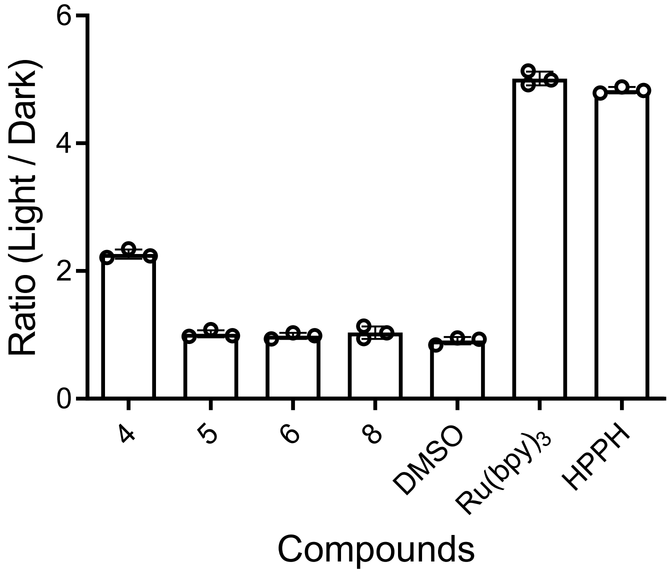

Supplementary Figure 27. Determination of irradiation-induced $^{1}O_2$ production with 660 nm light (1 hr = 58.7 J/cm^2) using the Singlet Oxygen Sensor Green assay. The error bars correspond to the standard deviation of the three replicates. Compounds **5–8** did not produced $^{1}O_2$ in contrast to the control compounds HPPH (Photochlor, 10 μM) and Ru(bpy)$_3$ Note: Ru(bpy)$_3$ was tested at 5 μM concentration with an indigo LED light source for 1 min (29.1 J/cm^2, 450 nm) as it does not absorb red light.

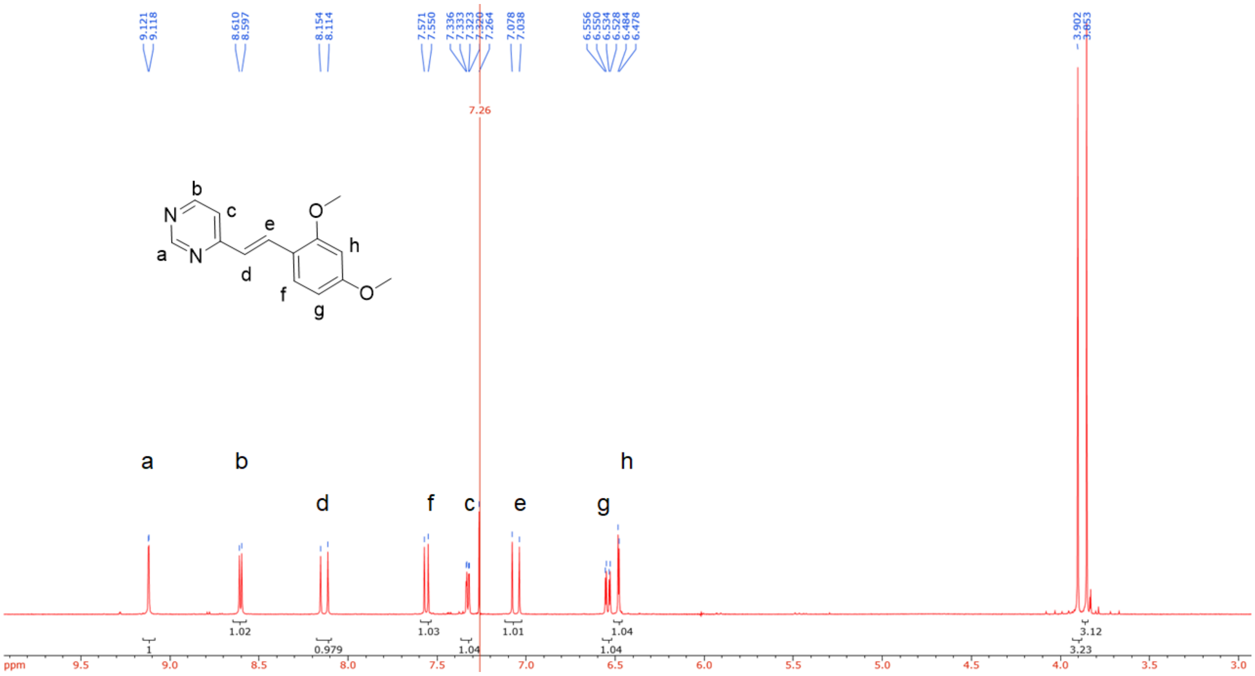

Supplementary Figure 28. ^{1}H NMR of **2** in $CDCl_3$.

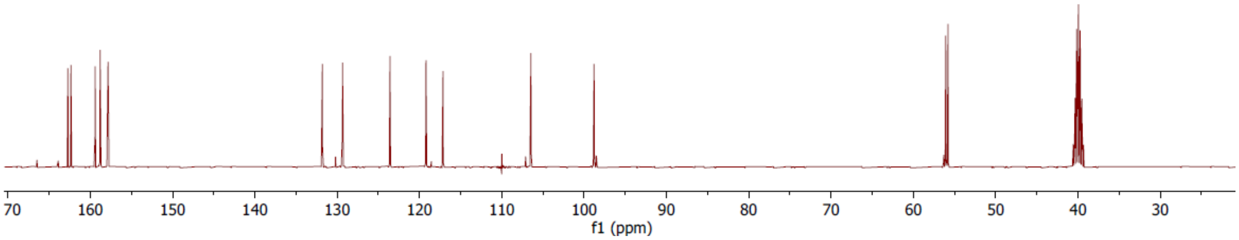

Supplementary Figure 29. ^{13}C NMR of **2** in DMSO.

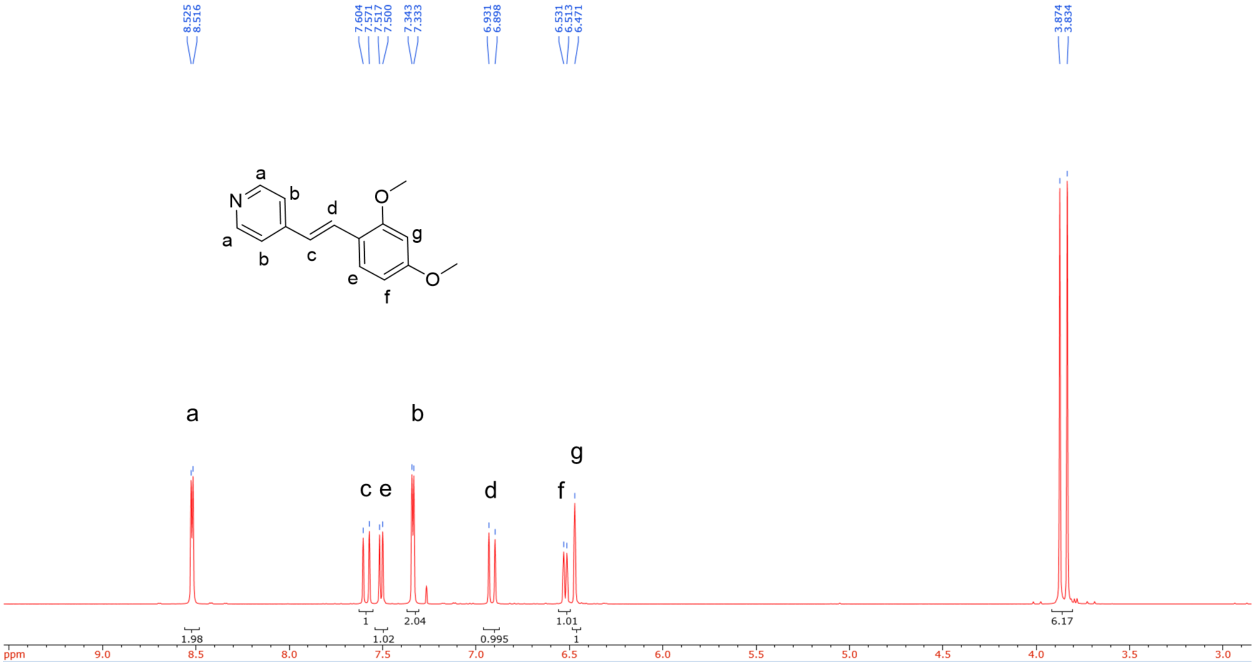

Supplementary Figure 30. ^{1}H NMR of **3** in $CDCl_3$.

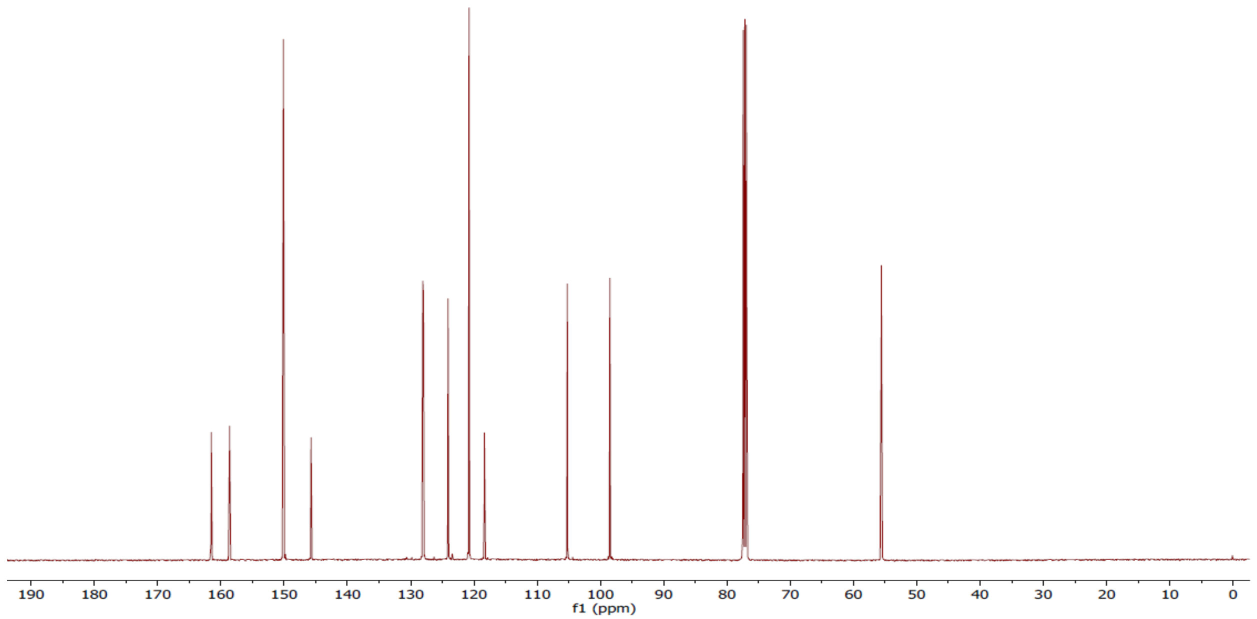

Supplementary Figure 31. ^{13}C NMR of **3** in $CDCl_3$.

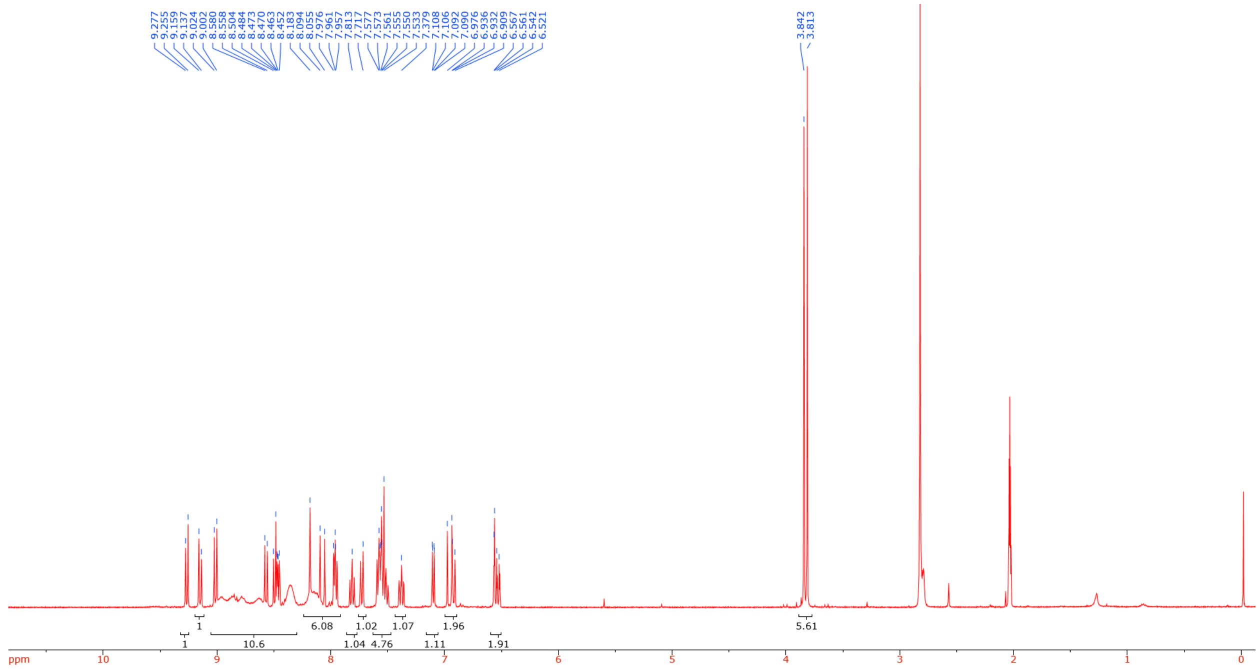

Supplementary Figure 32. ^{1}H NMR of **4** in CD_3CN.

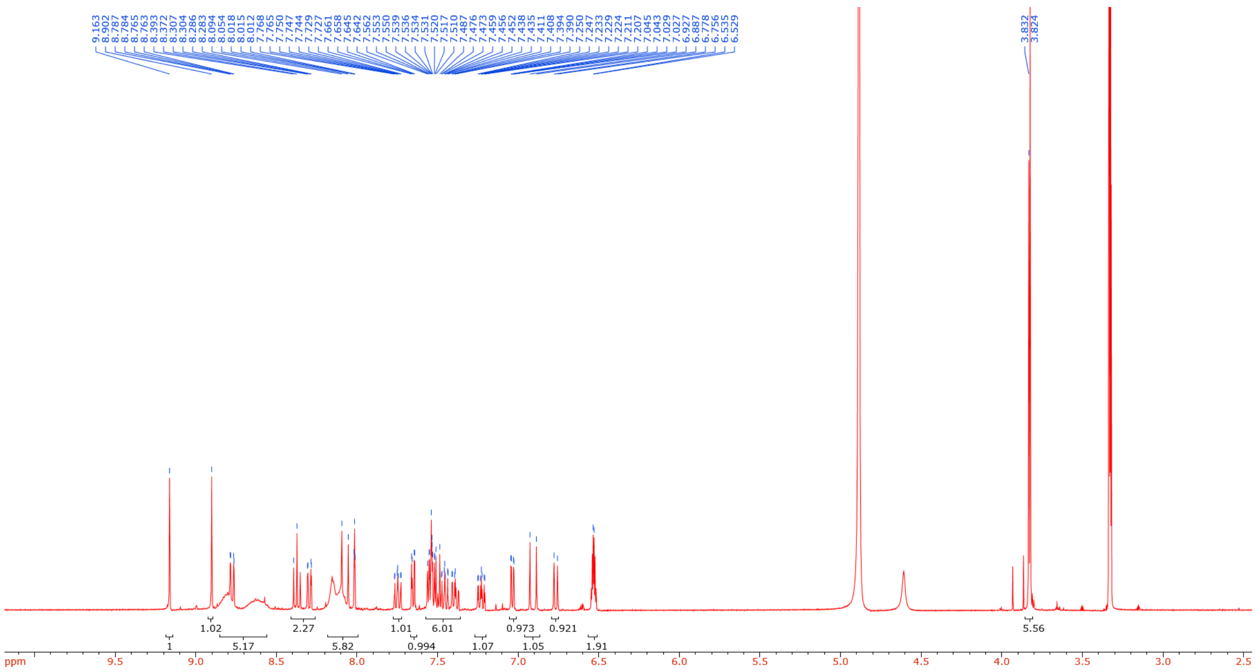

Supplementary Figure 33. ^{1}H NMR of **5** in CD_3OD.

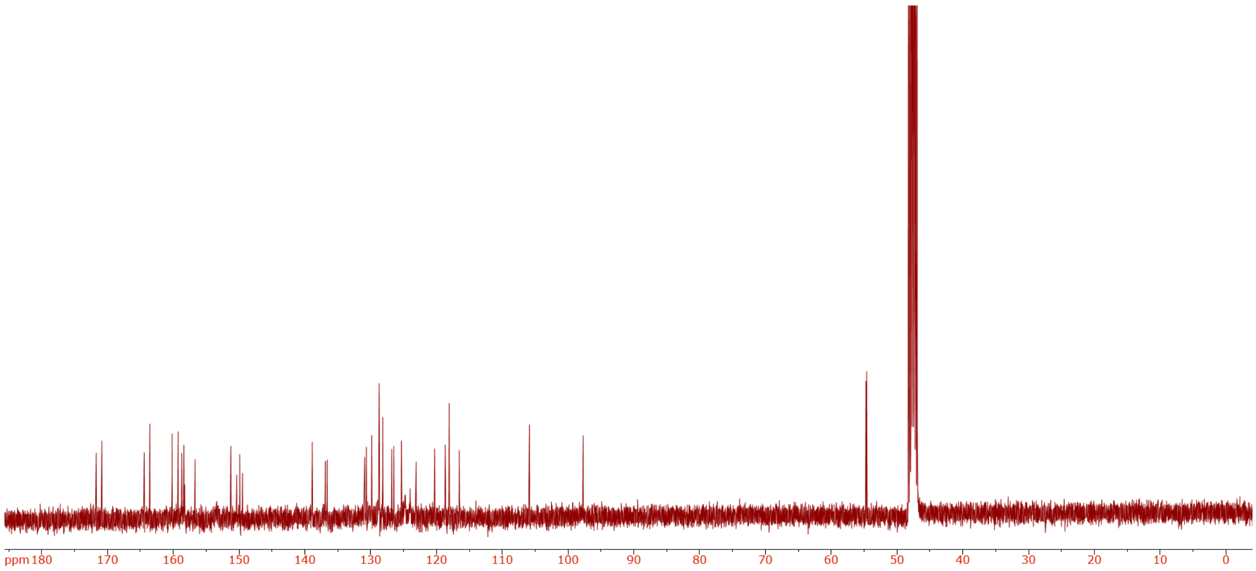

Supplementary Figure 34. ^{13}C NMR of **5** in CD_3OD.

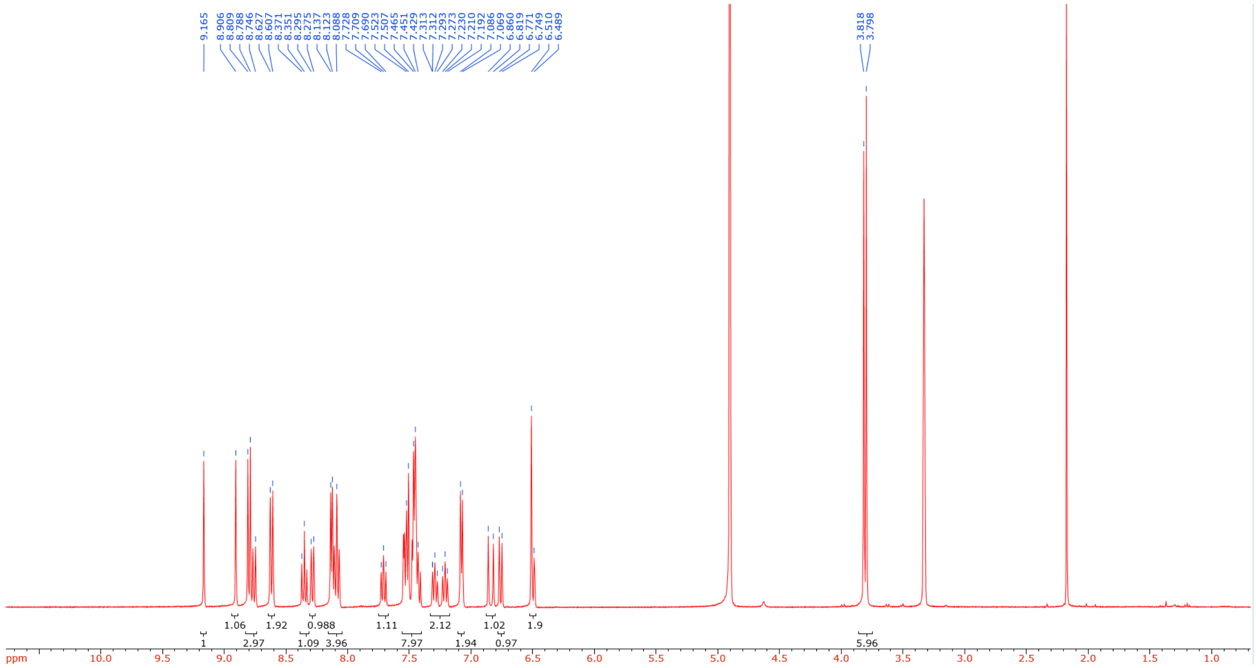

Supplementary Figure 35. ^{1}H NMR of **6** in MeOD.

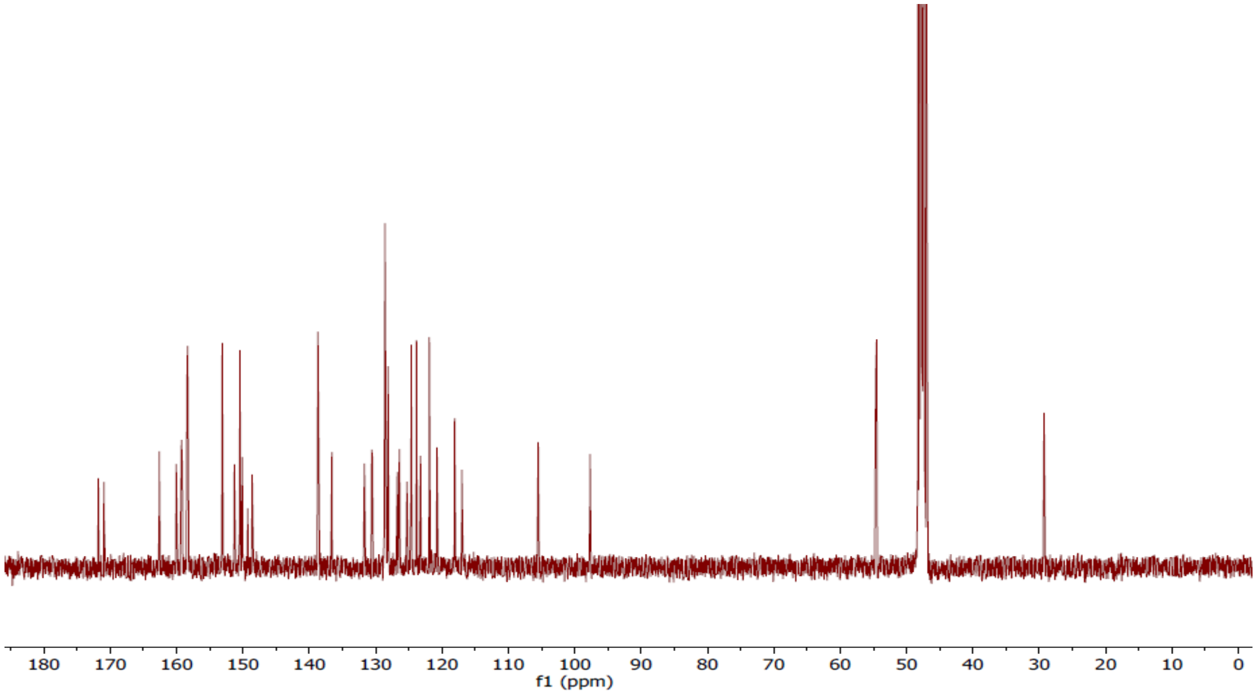

Supplementary Figure 36. ^{13}C NMR of **6** in MeOD.

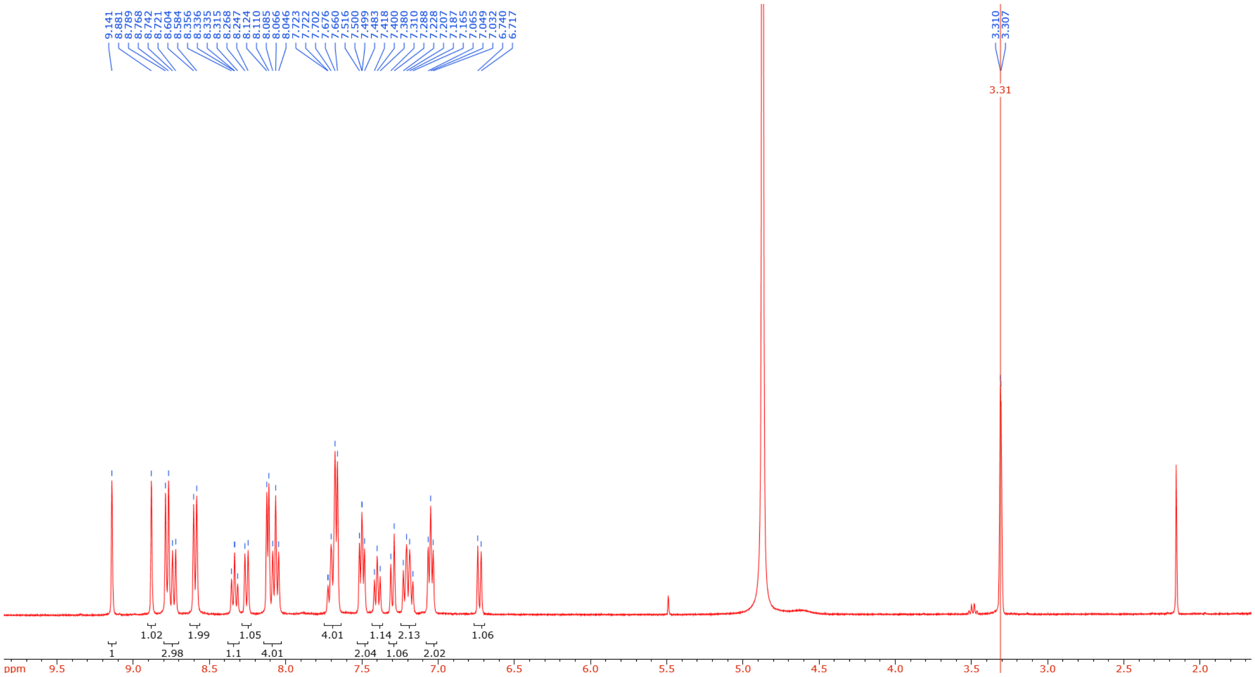

Supplementary Figure 37. ^{1}H NMR of **[Ru(tpy)(bca)(pyridine)]** (**8**) in MeOD.

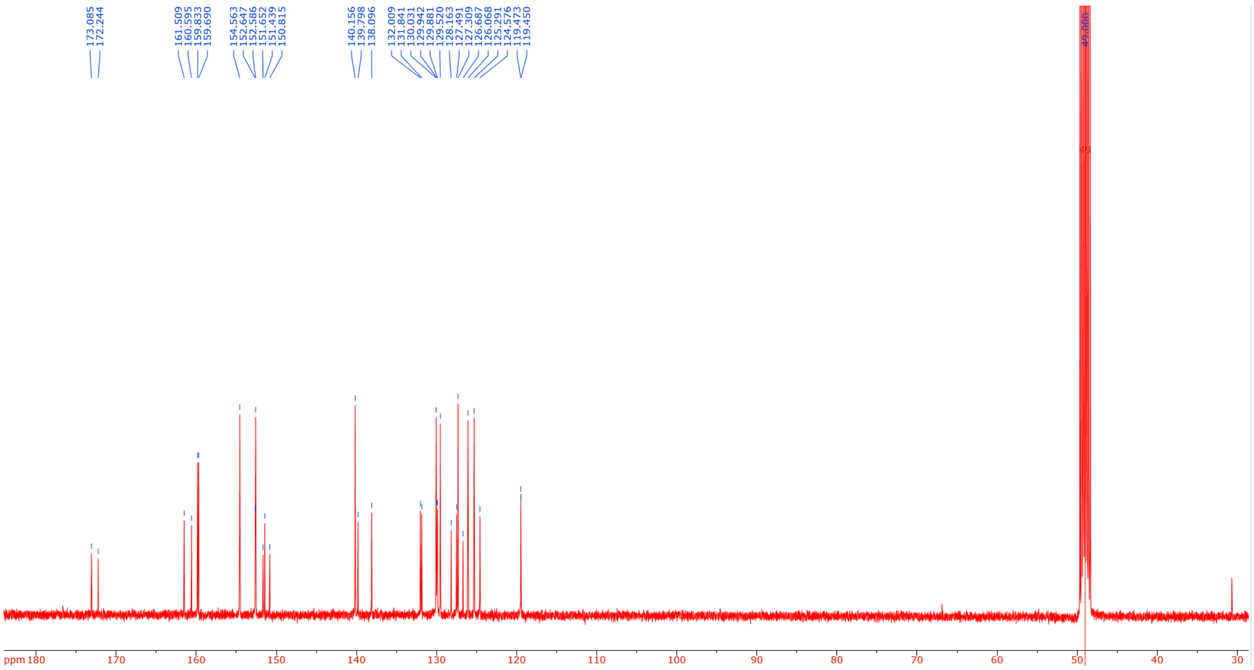

Supplementary Figure 38. ^{13}C NMR of **[Ru(tpy)(bca)(pyridine)]** (**8**) in MeOD.

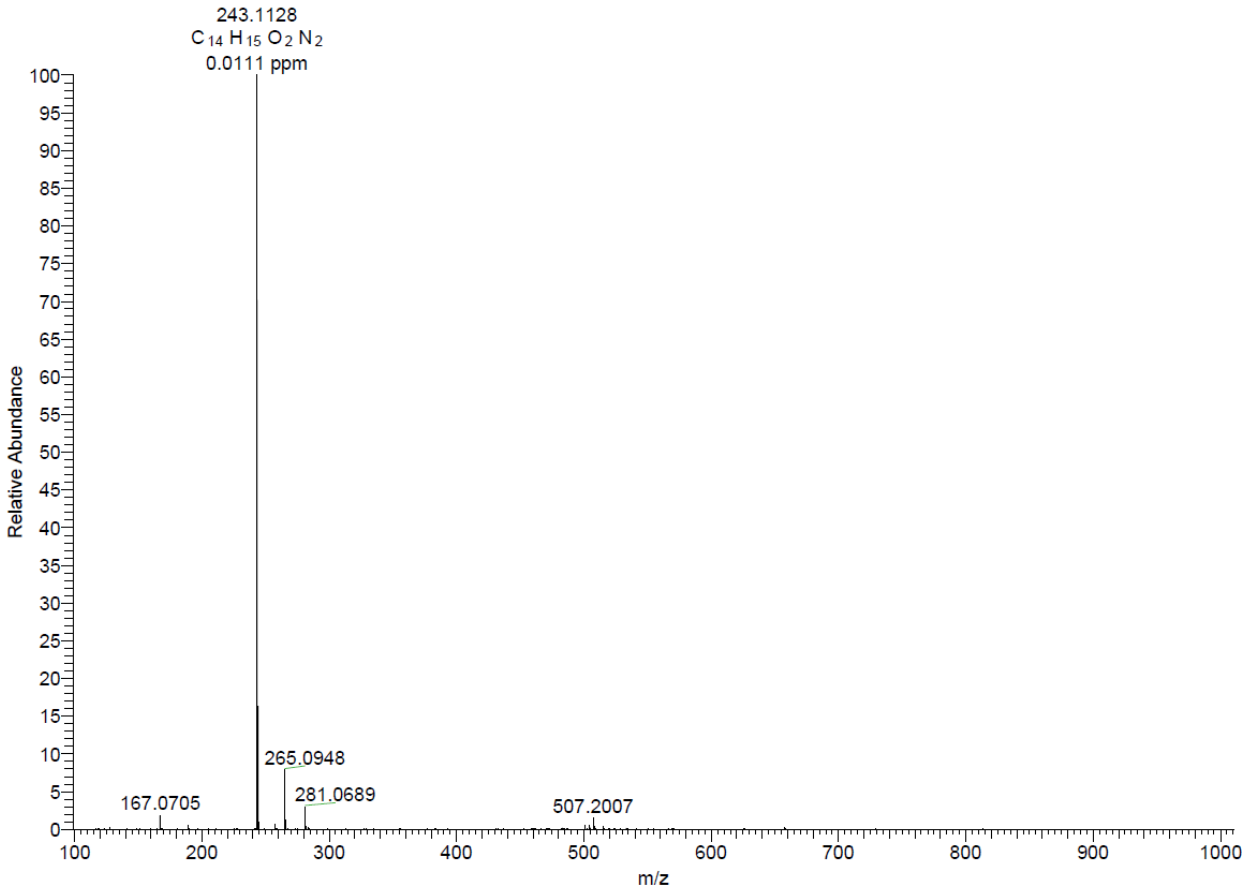

Supplementary Figure 39. ESI-MS of compound **2** calcd for $C_{14}H_{14}N_2O_2$ $[MH]^+$ 243.1134, found 243.1128 $[MH]^+$.

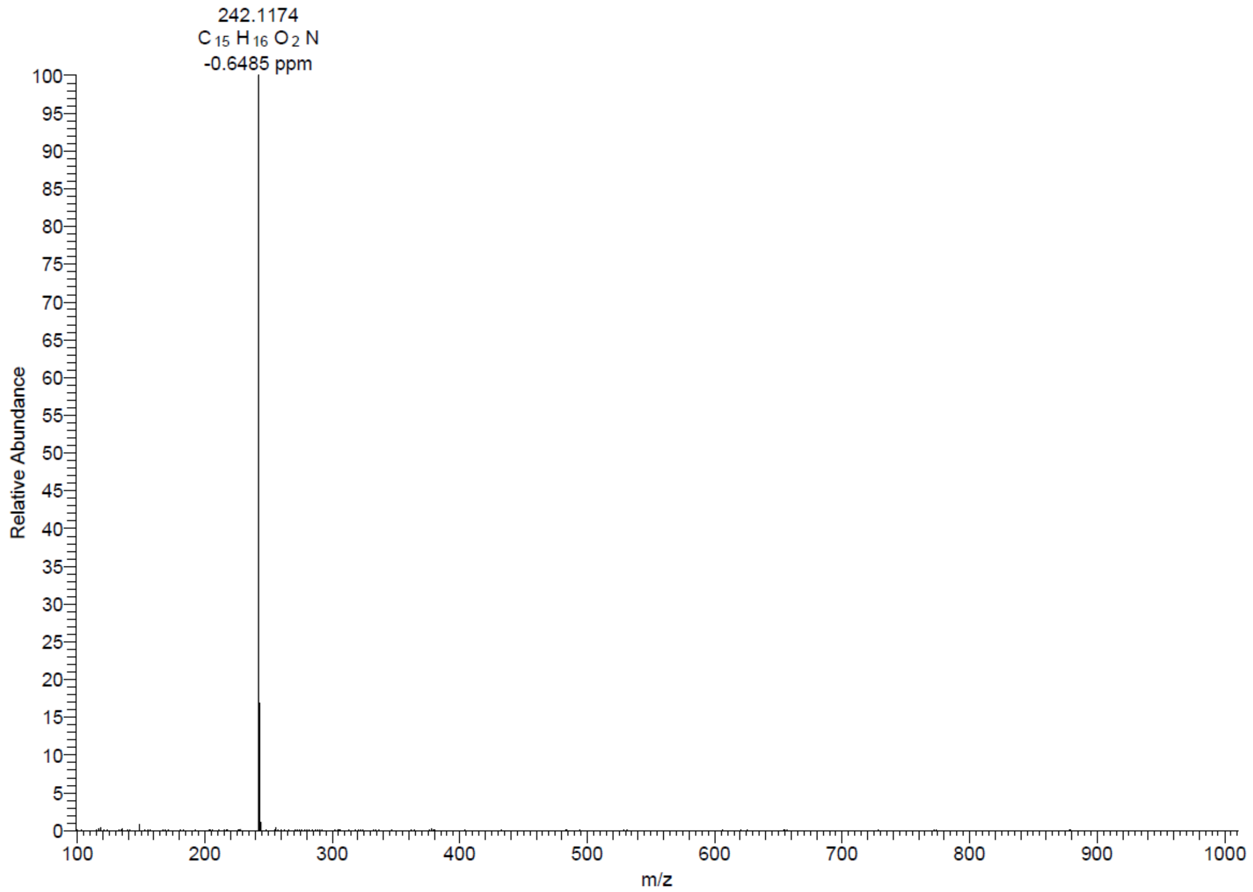

Supplementary Figure 40. ESI-MS of compound **3** calcd for $C_{15}H_{15}NO_2$ $[MH]^+$ 242.1181, found 242.1174 $[MH]^+$.

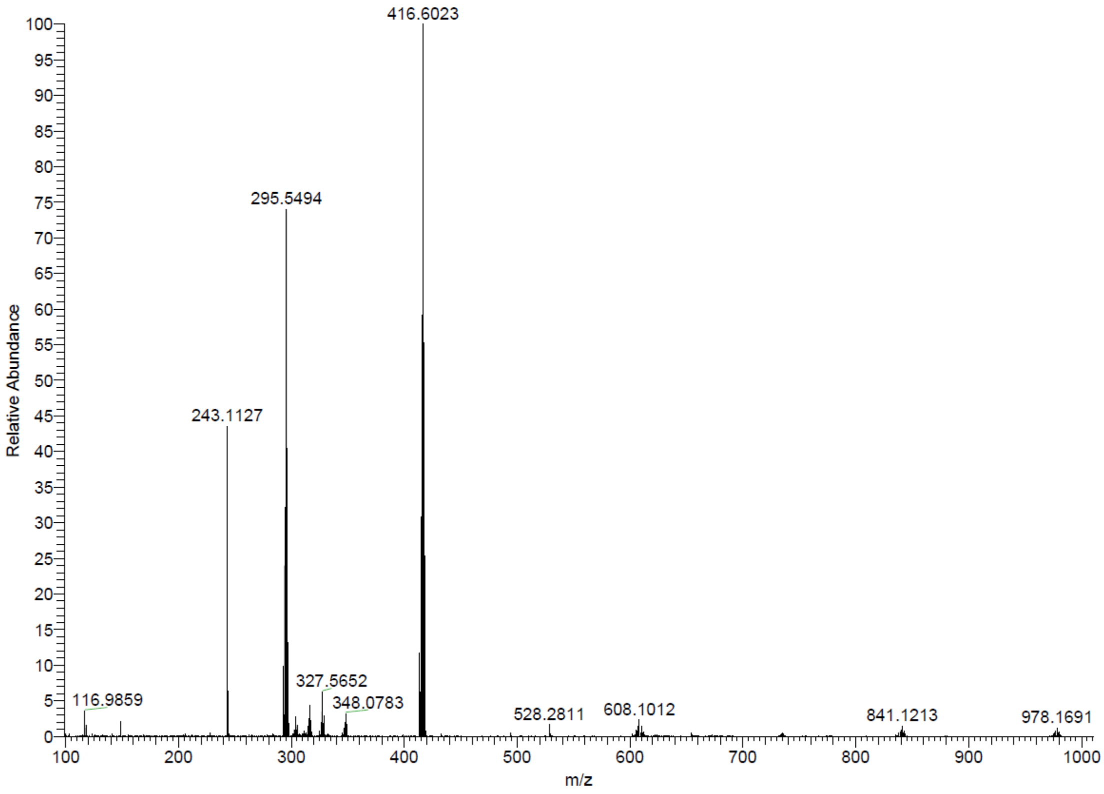

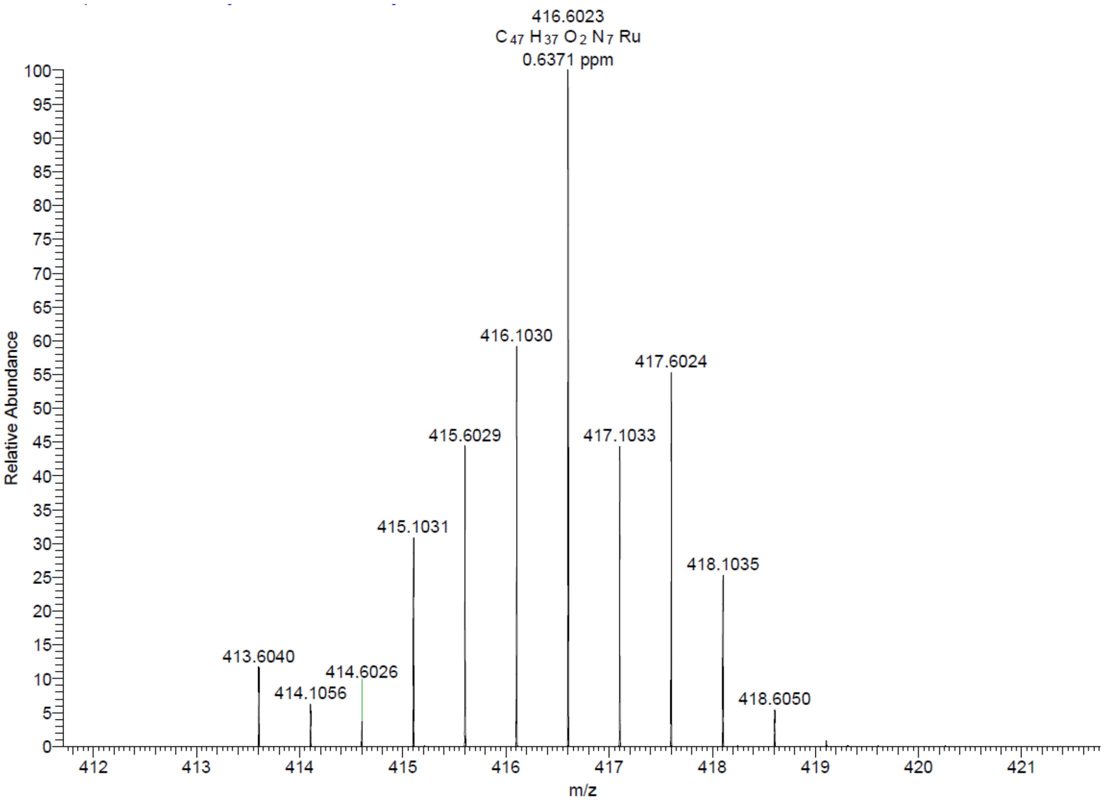

Supplementary Figure 41. ESI-MS of compound **4** calcd for $C_{47}H_{37}N_7O_7Ru$ $[M]^{2+}$ 416.6026; found 416.6023 $[M]^{2+}$.

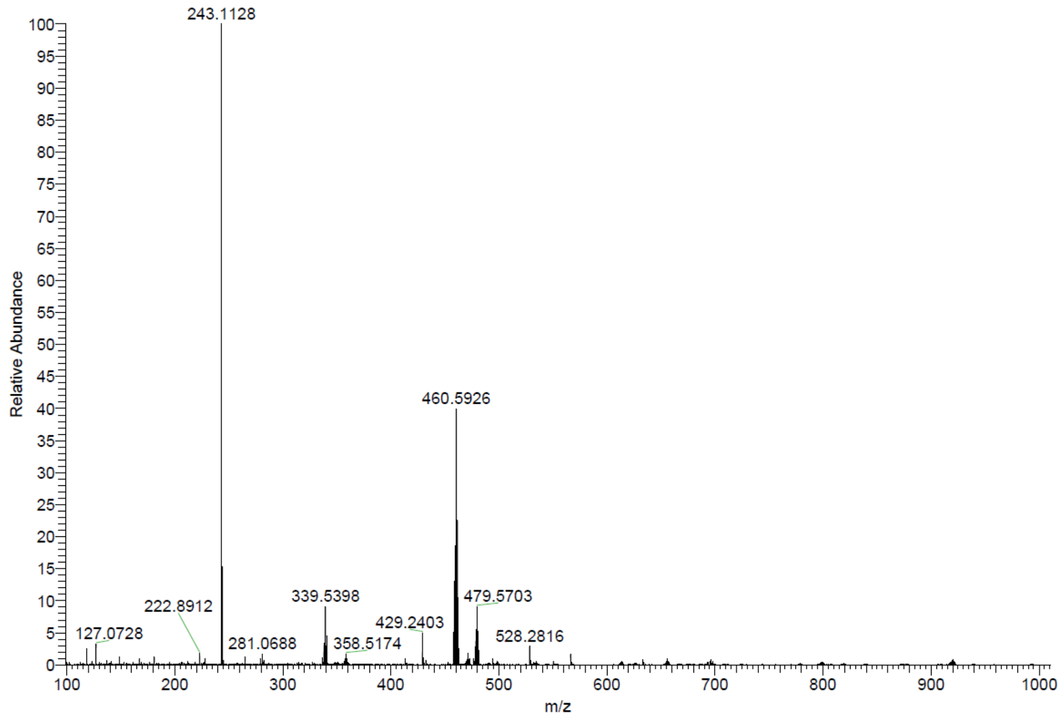

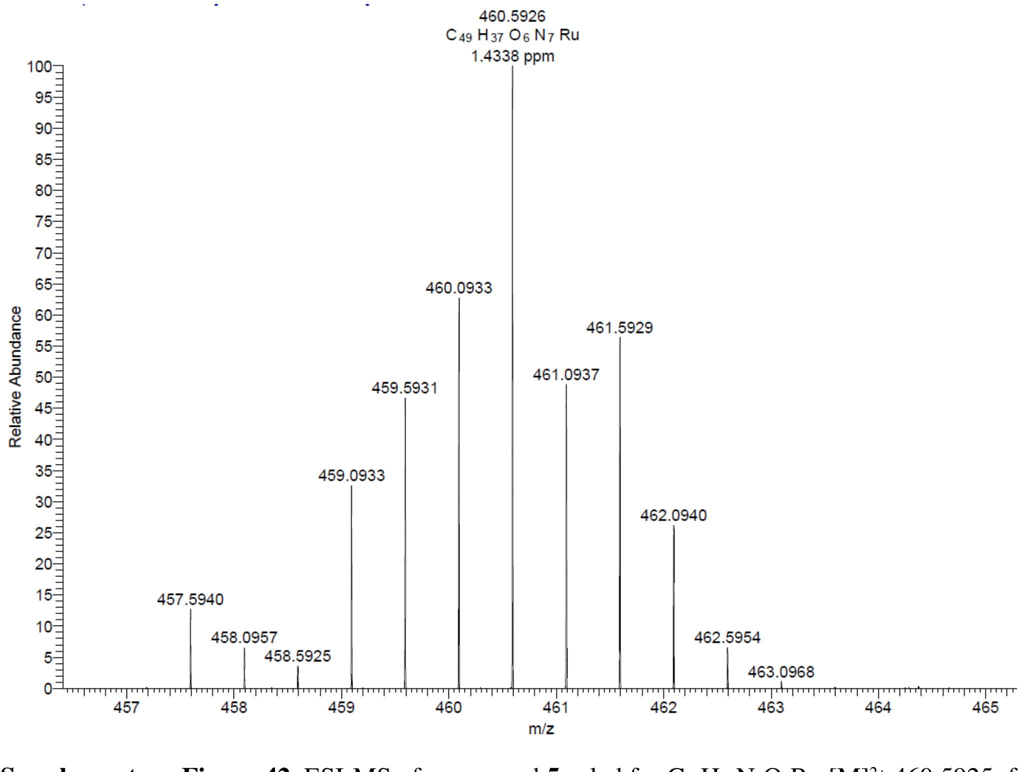

Supplementary Figure 42. ESI-MS of compound **5** calcd for $C_{49}H_{37}N_7O_6Ru$ $[M]^{2+}$ 460.5925; found 460.5926 $[M]^{2+}$.

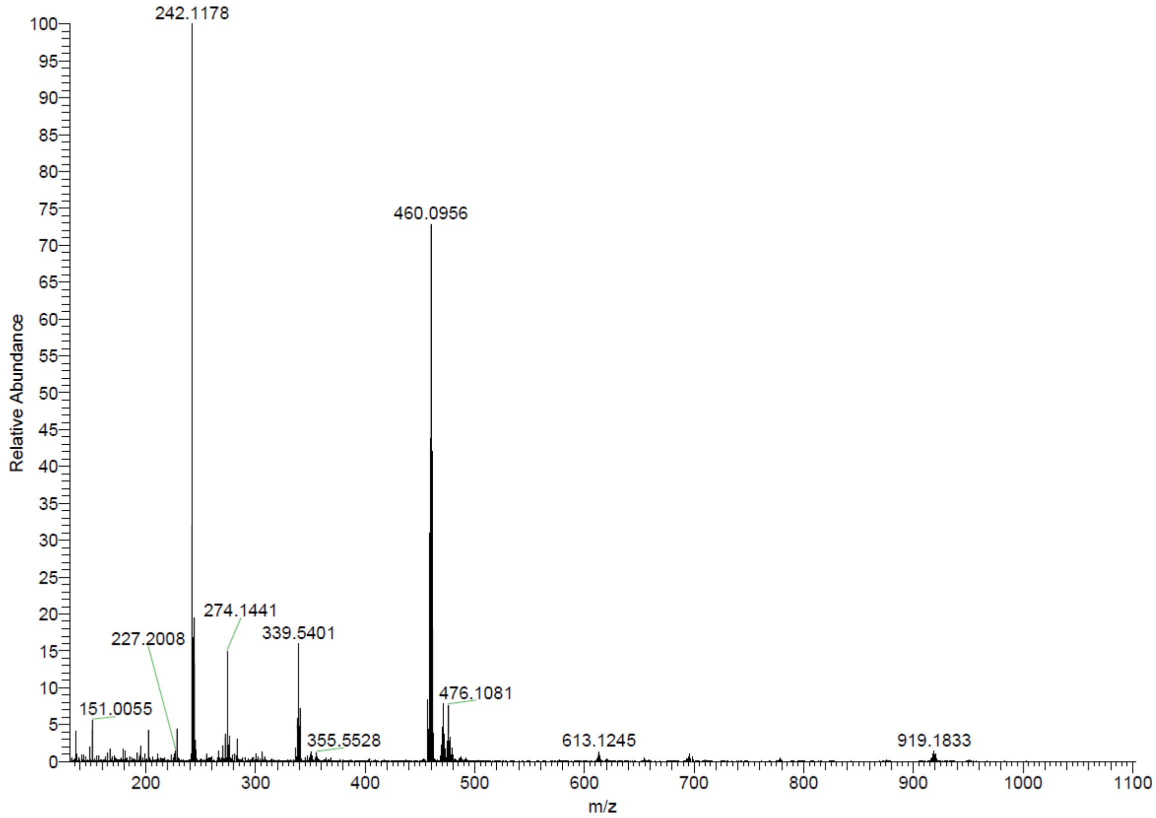

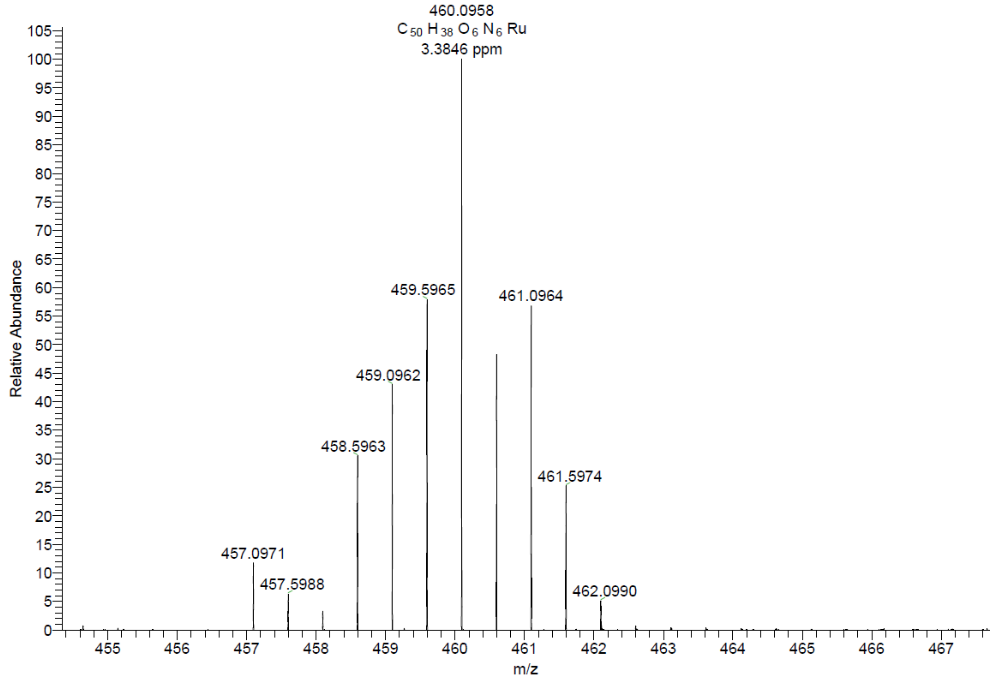

Supplementary Figure 43. ESI-MS of compound **6** calcd for $C_{50}H_{38}N_6O_6Ru$ $[M]^{2+}$ 460.0948; found 460.0958 $[M]^{2+}$.

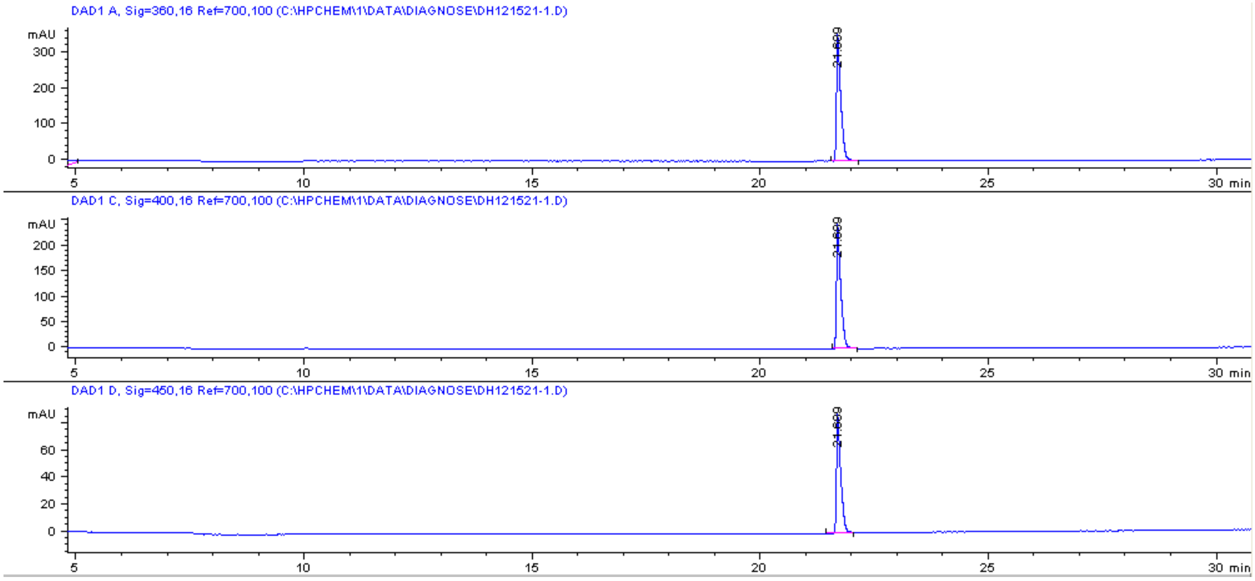

Supplementary Figure 44. HPLC chromatograms of **6** with detection wavelengths of 360 nm (top), 400 nm (middle) and 450 nm (bottom). The wavelengths were chosen to ensure sensitive detection of all species: 360 nm is the single wavelength with the maximum absorbance for all compounds in the mixture, **3, 6,** and **7** (see Fig. S11); 400 nm is the point of maximum absorbance of **3** when protonated (Fig 3a); 450 nm was used to ensure detection of other Ru(II) species.

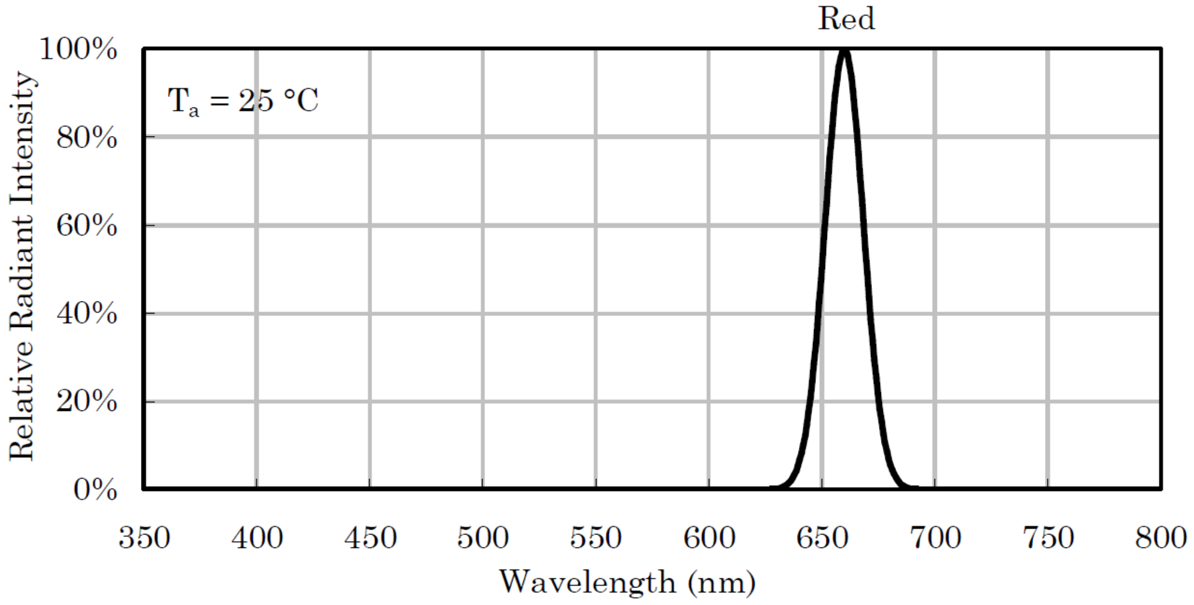

Supplementary Figure 45. Relative Intensity of T-1 3/4 (5mm) Solid State Lamp (120 LED Array Red from Elixa includes 120 Solid State Lamps). Wavelength of peak emission λ_P = 660 nm, spectral line full width at half-maximum $\Delta\lambda$ = 20 nm.

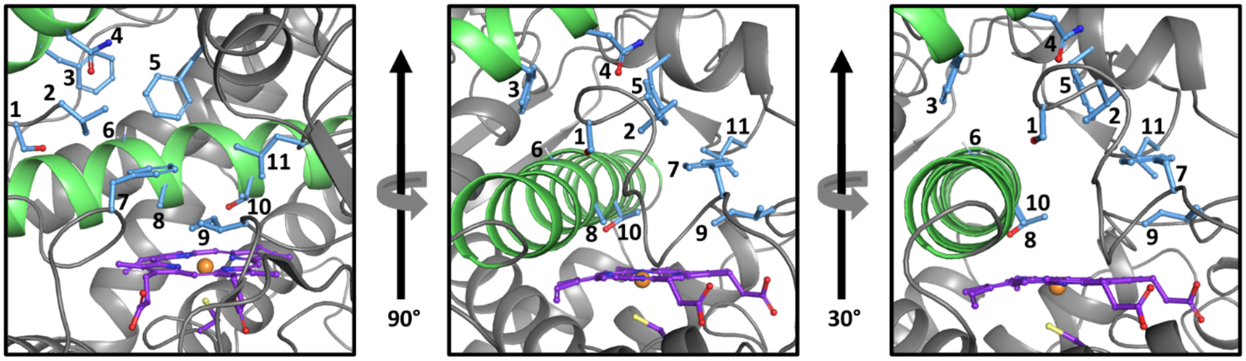

Supplementary Figure 46. Relative locations of residues (blue) with contacts enumerated in **Supplementary Table 1**. The I helix (center) and G helix (top) are shown as green ribbons. The heme and its ligating Cys470 are shown as purple sticks 1: Ser127, 2: Val126, 3: Phe268, 4: Asn265, 5: Phe231, 6: Gly329, 7: Phe134, 8: Ala330, 9: Ile399, 10: Thr334, 11: Leu509.

Supplementary Tables

Supplementary Table 1. Prevalence of select protein-ligand contacts.

Residue	**2** (% of frames interaction is present)	**3** (% of frames interaction is present)
Val126	0.2	24.4
Ser127	5.2	21.7
Phe134	11.0	27.8
Phe231	87.3	69.8
Asn265	12.8	17.3
Phe268	51.7	44.9
Gly329	42.6	5.0
Ala330	41.8	67.7
Thr334	70.3	0
Ile399	62.2	28.4
Leu509	24.3	24

Supplementary Table 2. IC_{50} values for inhibitors in CYP1B1 variants. The ratio is the IC_{50} (mutant)/ IC_{50} (WT).

**Compound**	**IC_{50} WT**	**S127A**	**Ratio**	**F134L**	**Ratio**	**S269A**	**Ratio**	**Q332E**	**Ratio**	**D333N**	**Ratio**
ANF	0.14	0.009	**0.064**	0.07	**0.5**	0.14	**1**	1.127	**8**	4.4	**31**
**1**	0.083	0.0025	**0.030**	0.87	**10**	0.18	**2**	0.2979	**3.6**	8.1	**98**
**2**	0.076	3.74	**50**	0.24	**3**	0.073	**1**	0.4024	**5.3**	5.0	**66**
**3**	0.00031	0.048	**160**	0.015	**50**	0.0021	**7**	0.0508	**169**	0.7	**2333**

Supplementary Table 3. Kinetic solubility test for compounds **4–6**.

	Volume, DMSO stock, µL	C, DMSO stock, mM	Volume Opti-MEM, µL	λ_{Abs}, nm	Abs1	Abs2	Abs3	C1, µM	C2, µM	C3, µM	C average, µM	SD, µM
**4**	14	10	700	535	0.441	0.44	0.395	80.9	80.7	72.5	**78.0**	**4.8**
**5**	14	10	700	540	0.357	0.348	0.355	113.3	110.0	113.0	**112.2**	**1.5**
**6**	14	10	700	550	0.112	0.127	0.126	33.9	38.5	38.2	**36.9**	**2.5**

Supplementary Table 4. Stability of compounds in human liver microsomes.

Compound	**R2**	**$T_{1/2}$, min**	**$CL_{int(mic)}$, µL/min/mg**	**$CL_{int(liver)}$, mL/min/kg**	**Remaining, % (T=60min)**	**Remaining, % (NCF=60min)**
**1**	0.9181	17.072	81.186	73.0674	8.5	77.1
**2**	0.9758	6.9	200.863	180.7767	0.4	96.5
**3**	0.9756	8.066	171.826	154.6434	0.6	92.1
[Os(bpy)$_2$(**2**)Cl]Cl	0.8649	>145	<9.6	<8.6	78.4	107
Testosterone	0.983	12.319	112.507	101.2563	3.6	82.5
Diclofenac	0.9995	3.849	360.049	324.0441	0.0	86.4
Propafenone	0.9334	5.317	260.69	234.621	0.0	92.4

Supplementary Table 5. Stability of compounds in rat liver microsomes.

Compound	**R2**	**$T_{1/2}$, min**	**$CL_{int(mic)}$, µL/min/mg**	**$CL_{int(liver)}$, mL/min/kg**	**Remaining, % (T=60min)**	**Remaining, % (NCF=60min)**
**1**	0.9622	14.634	94.709	170.4762	5.8	63.7
**2**	0.9835	6.754	205.21	369.378	0.3	47.1
**3**	0.9569	10.372	133.629	240.5322	1.4	108.6
[Os(bpy)$_2$(**2**)Cl]Cl	0.935	81.9	16.9	30.5	56.5	91.8
Testosterone	0.9451	2.074	668.429	1203.1722	0.8	86.5
Diclofenac	0.9965	17.632	78.607	141.4926	9.0	87.7
Propafenone	0.9901	1.909	725.873	1306.5714	0.0	103.2

Supplementary Table 6. Stability of compounds in mouse liver microsomes.

Compound	**R2**	**$T_{1/2}$, min**	**$CL_{int(mic)}$, µL/min/mg**	**$CL_{int(liver)}$, mL/min/kg**	**Remaining, % (T=60min)**	**Remaining, % (NCF=60min)**
**1**	0.9937	2.588	535.489	2120.53644	3.5	83.0
**2**	0.9889	1.514	915.359	3624.82164	0.1	111.5
**3**	0.9919	2.888	479.929	1900.51884	0.1	137.9
[Os(bpy)$_2$(**2**)Cl]Cl	0.5657	>145	<9.6	<38.0	71.8	75.9
Testosterone	0.9998	2.787	497.271	1969.19316	2.3	91.3
Diclofenac	0.9748	30.888	44.871	177.68916	27.9	95.4
Propafenone	0.9916	1.422	974.85	3860.406	0.0	111.8

Notes:

NCF: abbreviation of no co-factor. No NADPH is added to NCF samples (replaced by buffer) during the 60 minute incubation. If the NCF remaining is less than 60%, then possibly non-NADPH dependent metabolism occurs

R^2: correlation coefficient of the linear regression for the determination of kinetic constant

$T_{1/2}$: half life

$CL_{int(mic)}$: intrinsic clearance

$CL_{int(mic)}$ = 0.693/$T_{1/2}$/mg microsome protein per mL

$CL_{int(liver)}$ = $CL_{int(mic)}$ * mg microsomal protein/g liver weight * g liver weight/kg body weight

Species	Liver Weight (g/kg Body Weight)	Hepatic Blood Flow (Q_h) (mL/min/kg)	Microsomal Protein (mg/g liver weight)
Mouse	88	90	
Rat	40	55.2	
Dog	32	30.9	45
Monkey	30	43.6	
Human	20	20.7	

Supplementary Table 7. Quantum yields of photosubstitution in different solvents.

Compound	$\Phi_{(PS)}$ 5% DMSO in H_2O[a]	$\Phi_{(PS)}$ Opti-MEM[a]	$\Phi_{(PS)}$ CH_3OH
**4**	0.055 ± 0.003	0.041 ± 0.006	
**5**	0.02 ± 0.003	0.013 ± 0.002	
**6**[b]	0.00043 ± 0.00005		0.002 ± 0.00005
**8**	0.0059 ± 0.0005	0.004 ± 0.00005	

[a] Quantum yield for photosubstitution, Φ_{PS}, in 5% DMSO in H_2O and Opti-MEM, calculated by optical approach (Fig. S8-10). [b] Φ_{PS} in 5% DMSO in H_2O, determined by HPLC approach. HPLC was used due to overlap in absorbance profiles for **6** and its photochemical product (Fig. S7).

Supplementary Table 8. Lipinski's rule of five.

	MW	cLogP ChemDraw / SwissADME	HBD	HBA
**1**	300.35	3.7 / 3.7	0	4
**2**	242.28	2.5/ 2.4	0	4
**3**	241.29	2.6 / 2.9	0	3
**4**	832.93	-0.6 [a]	0	9
**5**	920.95	-0.5 [a]	2	13
**6**	919.96	0.2 [a]	2	12

[a] log P was calculated by taking the logarithm of the ratio of the MLCT absorbance of each complex (**4-6**) in octanol to the corresponding absorbance in H_2O.

References

1. Lameijer, L. N. *et al*. A Red-Light-Activated Ruthenium-Caged NAMPT Inhibitor Remains Phototoxic in Hypoxic Cancer Cells. *Angew. Chem. Int. Ed. Engl.* **56**, 11549-11553, doi:10.1002/anie.201703890 (2017).
