# Supplementary material for: Ru(II) photocages enable precise control over enzyme activity with red light
Source: Nat Commun. 2022 Jun 25;13:3636. doi: 10.1038/s41467-022-31269-5 (PMC9233675; doi:10.1038/s41467-022-31269-5)
Supplement: Supplementary file 3 — Reporting Summary [file 41467_2022_31269_MOESM3_ESM.pdf]

nature portfolio

Corresponding author(s): Edith Glazer, David Heidary

Last updated by author(s): May 25, 2022

Reporting Summary

Nature Portfolio wishes to improve the reproducibility of the work that we publish. This form provides structure for consistency and transparency in reporting. For further information on Nature Portfolio policies, see our Editorial Policies and the Editorial Policy Checklist.

Statistics

For all statistical analyses, confirm that the following items are present in the figure legend, table legend, main text, or Methods section.

n/a	Confirmed	
[ ]	[x]	The exact sample size (*n*) for each experimental group/condition, given as a discrete number and unit of measurement
[ ]	[x]	A statement on whether measurements were taken from distinct samples or whether the same sample was measured repeatedly
[x]	[ ]	The statistical test(s) used AND whether they are one- or two-sided<br>*Only common tests should be described solely by name; describe more complex techniques in the Methods section.*
[ ]	[x]	A description of all covariates tested
[ ]	[x]	A description of any assumptions or corrections, such as tests of normality and adjustment for multiple comparisons
[ ]	[x]	A full description of the statistical parameters including central tendency (e.g. means) or other basic estimates (e.g. regression coefficient) AND variation (e.g. standard deviation) or associated estimates of uncertainty (e.g. confidence intervals)
[x]	[ ]	For null hypothesis testing, the test statistic (e.g. *F*, *t*, *r*) with confidence intervals, effect sizes, degrees of freedom and *P* value noted<br>*Give P values as exact values whenever suitable.*
[x]	[ ]	For Bayesian analysis, information on the choice of priors and Markov chain Monte Carlo settings
[x]	[ ]	For hierarchical and complex designs, identification of the appropriate level for tests and full reporting of outcomes
[x]	[ ]	Estimates of effect sizes (e.g. Cohen's *d*, Pearson's *r*), indicating how they were calculated

Our web collection on statistics for biologists contains articles on many of the points above.

Software and code

Policy information about availability of computer code

Data collection	Tecan Magellan Standard 7.1, Jasco CD Spectra Manager v. 2, Agilent ChemStation for HPLC Rev.B.01.03[204] and UV/Vis A.09.0x, Desmond Molecular Dynamics System (Schrodinger).
Data analysis	Prism V6.

For manuscripts utilizing custom algorithms or software that are central to the research but not yet described in published literature, software must be made available to editors and reviewers. We strongly encourage code deposition in a community repository (e.g. GitHub). See the Nature Portfolio guidelines for submitting code & software for further information.

Data

Policy information about availability of data

All manuscripts must include a data availability statement. This statement should provide the following information, where applicable:

- Accession codes, unique identifiers, or web links for publicly available datasets
- A description of any restrictions on data availability
- For clinical datasets or third party data, please ensure that the statement adheres to our policy

The data that support the findings of this study are avail-able within the paper or its supplementary information. Source data are provided with this paper, or upon request from the corresponding author.

Field-specific reporting

Please select the one below that is the best fit for your research. If you are not sure, read the appropriate sections before making your selection.

- [x] Life sciences
- [ ] Behavioural & social sciences
- [ ] Ecological, evolutionary & environmental sciences

For a reference copy of the document with all sections, see nature.com/documents/nr-reporting-summary-flat.pdf

Life sciences study design

All studies must disclose on these points even when the disclosure is negative.

Sample size	All screening data was performed in cell lines or with commercial mirosomes, and the sample size was validated by the quality of assay response (Z'). For cell lines, cells were plated at 40,000 cells/well. For all experiments, a minimum of n=3 experimental replicates were used.
Data exclusions	No data was excluded.
Replication	All data was replicated by a minimum of two repeated experiment, with a minimum of replication over period of weeks.
Randomization	As the data came from screening, there was no randomization. Covariates were controlled as the experiments were performed for many compounds, hundreds of times, and control compounds gave consistent results.
Blinding	The biological testing was done by a person who only was provided compound codes, so they did not know the identity or nature of the compounds.

Reporting for specific materials, systems and methods

We require information from authors about some types of materials, experimental systems and methods used in many studies. Here, indicate whether each material, system or method listed is relevant to your study. If you are not sure if a list item applies to your research, read the appropriate section before selecting a response.

Materials & experimental systems

n/a	Involved in the study	
x		Antibodies
	x	Eukaryotic cell lines
x		Palaeontology and archaeology
x		Animals and other organisms
x		Human research participants
x		Clinical data
x		Dual use research of concern

Methods

n/a	Involved in the study	
x		ChIP-seq
x		Flow cytometry
x		MRI-based neuroimaging

Eukaryotic cell lines

Policy information about cell lines

Cell line source(s)	HEK T-Rex cell line was obtained from Thermo Fisher.
Authentication	The cell lines were not independently authenticated.
Mycoplasma contamination	The cell lines tested negative by the EZ-PCR mycoplasma test kit (Sartorius)
Commonly misidentified lines (See ICLAC register)	No commonly misidentified cell lines were used.
